# *Rhizobium* symbiosis improves amino acid and secondary metabolite biosynthesis of tungsten-stressed soybean (*Glycine max*)

**DOI:** 10.3389/fpls.2024.1355136

**Published:** 2024-04-02

**Authors:** Julian Preiner, Irene Steccari, Eva Oburger, Stefanie Wienkoop

**Affiliations:** ^1^Molecular Systems Biology Unit, Department of Functional and Evolutionary Ecology, University of Vienna, Vienna, Austria; ^2^Department of Forest and Soil Sciences, Institute of Soil Research, University of Natural Resources and Life Sciences Vienna, Tulln, Austria

**Keywords:** symbiosis, abiotic stress, tungsten, metabolomics, leguminous plants

## Abstract

The industrially important transition metal tungsten (W) shares certain chemical properties with the essential plant micronutrient molybdenum and inhibits the activity of molybdoenzymes such as nitrate reductase, impacting plant growth. Furthermore, tungsten appears to interfere with metabolic processes on a much wider scale and to trigger common heavy metal stress response mechanisms. We have previously found evidence that the tungsten stress response of soybeans (*Glycine max*) grown with symbiotically associated N_2_-fixing rhizobia (*Bradyrhizobium japonicum*) differs from that observed in nitrogen-fertilized soy plants. This study aimed to investigate how association with symbiotic rhizobia affects the primary and secondary metabolite profiles of tungsten-stressed soybean and whether changes in metabolite composition enhance the plant’s resilience to tungsten. This comprehensive metabolomic and proteomic study presents further evidence that the tungsten-stress response of soybean plants is shaped by associated rhizobia. Symbiotically grown plants (N fix) were able to significantly increase the synthesis of an array of protective compounds such as phenols, polyamines, gluconic acid, and amino acids such as proline. This resulted in a higher antioxidant capacity, reduced root-to-shoot translocation of tungsten, and, potentially, also enhanced resilience of N fix plants compared to non-symbiotic counterparts (N fed). Taken together, our study revealed a symbiosis-specific metabolic readjustment in tungsten-stressed soybean plants and contributed to a deeper understanding of the mechanisms involved in the rhizobium-induced systemic resistance in response to heavy metals.

## 1 Introduction

The loss of arable soils due to built-up areas, climate change, and increasing environmental pollution with xenobiotic substances such as heavy metals (HM) and metalloids represents a major challenge for plant growth and productivity. To ensure future food safety, it is essential to better understand plant abiotic stress.

Heavy metals can be found across terrestrial ecosystems due to natural occurrence in soils and human activities such as mining, agriculture, waste disposal, or industrial production (DalCorso et al., 2013; Ovečka and Takáč, 2014). While heavy metals such as Cu, As, Al, Pb, Mn, Zn, and Cd have been extensively studied, the industrially important transition metal tungsten (W) has received comparatively little attention (Hossain and Komatsu, 2012; Kennedy et al., 2012). Although tungsten background concentrations in topsoil are relatively low, ranging from 0.1 to 2.7 mg kg^-1^, they have been reported to be considerably exceeded at contaminated sites (10–2,000 fold). Major sources of tungsten contamination include mining activity, discharge from tungsten-utilizing industries, disposal of tungsten-containing appliances and products, fertilizer application, and military activity (Koutsospyros et al., 2006; Kennedy et al., 2012).

Since tungsten shares several chemical properties regarding structure, electronegativity, ionic and atomic radii, electron configuration as well as range of oxidation states (-2 to +6) with the essential micronutrient molybdenum, it is essential to understand the effects of environmental contamination with tungsten on plant physiology, agricultural production, health, and ecosystems as a whole (Kletzin and Adams, 1996). Both molybdenum and tungsten primarily occur in environmental systems as oxyanions tungstate (WO_4_^2-^) and molybdate (MoO_4_^2-^), which are readily taken up by plants and are prospectively stored in vacuoles (Strigul et al., 2005; McGrath et al., 2010; Bittner, 2014). While molybdenum serves as a co-factor for key enzymes in the carbohydrate and nitrogen metabolism of all life forms, tungsten-containing enzymes have only been identified in prokaryotes (Kletzin and Adams, 1996; Bevers et al., 2009). Numerous studies have shown that the four plant molybdoenzymes (nitrate reductase, aldehyde oxidase, xanthine dehydrogenase, and sulfite oxidase) lose their catalytic function when tungsten replaces molybdenum in the co-factor (Harper and Nicholas, 1978; Deng et al., 1989; L’vov et al., 2002; Bevers et al., 2009; Xiong et al., 2012). More recently, studies explored the effect of tungsten on abscisic acid biosynthesis (Jiang et al., 2007) and showed that tungsten leads to reduction of plant growth and general toxicity symptoms (Strigul et al., 2005; Bamford et al., 2011) as well as induces programmed cell death and disruption of cortical microtubules in root cells (Adamakis et al., 2011). These findings indicate that tungsten has further cellular targets apart from molybdoenzymes and that, much like other heavy metals, it is phytotoxic on its own (Adamakis et al., 2012).

We previously demonstrated that tungsten triggers similar stress response mechanisms as other heavy metals but also found evidence that the molecular mechanisms of tungsten toxicity in plants differ depending on the mode of nitrogen (N) nutrition (Preiner et al., 2019). Additionally, we found that, the roots and nodules of symbiotically grown leguminous plants exhibit higher levels of proteins involved in hormone and flavonoid biosynthesis in the presence of tungsten (0.5 mM Na_2_WO_4_ corresponding to 91.9 mg W L^-1^), indicating a symbiosis-specific response to tungsten (Preiner et al., 2019). Furthermore, our results suggested that nitrogenase activity was not directly affected by W but rather indirectly by hampered nodule development as well as that symbiotic N_2_ fixation is able to partially compensate for the loss of N reductase activity, suggesting more efficient detoxification and compartmentalization in symbiotically grown plants (Oburger et al., 2018; Preiner et al., 2019). Rhizobacteria are known to influence plant growth and stress resistance *via* various mechanisms. Inoculation with so-called plant-growth-promoting rhizobacteria (PGPR) has been shown to increase the production of various primary and secondary metabolites, induce systemic resistance, enhance the bio-availability of plant nutrients as well as immobilize heavy metal ions (Shameer and Prasad, 2018; Shaffique et al., 2023). Rather recently, actinobacteria have also been shown to alleviate the phytotoxicity of tungsten nanoparticles on legumes (Selim et al., 2021).

Building on our previous findings, we here set out to investigate whether symbiosis with N_2_-fixing *B. japonicum* influences the primary metabolite profile and secondary metabolite production of tungsten-stressed soybean plants. Furthermore, we aimed to examine whether these symbiotically induced changes affect the plant’s tolerance to tungsten-induced toxicity. To evaluate the metabolic response of soybean plants with and without *Rhizobium* symbiosis, tungsten stress was applied to plants after 21 days of growth when symbiosis was fully established. As control group, nitrogen-fertilized plants were used.

To identify the key metabolic pathways involved in stress regulation, we adapted and refined a sequential extraction procedure to quantify and screen major stress-related marker metabolites prior to comprehensive mass spectrometric analysis of individual metabolites and proteins. Determining the quantitative changes in the levels of metabolites such as soluble sugars, phenols, flavonoids, tannins, and amino acids provides valuable insights into the overall changes and assists in the interpretation and analysis of untargeted omics data. Furthermore, it allows us to enrich shotgun molecular phenotyping via GC–MSMS and LC–MSMS by the additional quantification of pigments, phenolic compounds, starch as well as markers for antioxidant capacity, all from one sample.

## 2 Materials and methods

### 2.1 Plant culture and harvest

Soybean (*Glycine max* cv. Primus obtained from Die SAAT Austria) seeds were surface-sterilized with 5% sodium hypochlorite solution and sown into acid-washed pots containing an inert substrate mixture of vermiculite and perlite in a 5/3 (v/v) ratio to exclude contamination. Half of the seeds (N fix) were inoculated with 1:10 diluted commercial *Bradyrhizobium japonicum* inoculant (Radicin Soja Die SAAT Austria) and received 0.25 mM KNO_3_ for only 2 weeks; the other half served as non-symbiotic control (N fed) and was supplied with 10 mM KNO_3_. Seedlings were watered every other day with a modified half strength N-free Hoagland nutrient solution, pH 7.2 (Hoagland and Arnon, 1950). After 3 weeks, when nodules were formed and symbiosis was fully established, 0.5 mM tungstate (Na_2_WO_4_) was added to the nutrient solution. To investigate the effects of tungsten on N-fertilized and symbiotically grown soy plants, a zero tungsten control was realized for both N regimes. The plants were altogether grown in eight biological replicates, four of which were used for proteomic and metabolomic analysis and four for the assessment of physiological parameters and analysis of macro- and micronutrients. The plants were grown under controlled glasshouse conditions with a 12/12 light/dark cycle at 29/21°C and an approximate photosynthetic radiation of 400 µmol m^−1^s^−1^ at canopy level. After 2 weeks of tungsten exposure, chlorophyll fluorescence measurements were performed, and the plants were sampled. The roots, nodules, and leaves of four biological replicates were cut off using a stainless-steel razorblade, transferred into precooled 2-mL Eppendorf tubes, immediately put on liquid nitrogen (LN_2_) for metabolomic and proteomic analysis, and stored at −80°C. The remaining four replicates were harvested for acid digestion and ICP-OES analysis.

### 2.2 Chlorophyll fluorescence measurements

Chlorophyll fluorescence was measured on individual leaves with a mini-pam II/B (Heinz Walz GmbH). The plants were allowed to adapt in the dark for 15 min and then subjected to a rapid light curve (RLC) with increasing photosynthetic active radiation (PAR) intensities (0, 25, 45, 65, 90, 125, 191, 287, 422, 633, 827, 1,157, and 1,507 μmol m^−2^ s^−1^) in increments of 20 s each. The calculation of fluorescence ratio parameters was done with WinControl -3 software (Heinz Walz GmbH) provided by the manufacturers of the pulse-amplitude modulated (PAM) fluorometer.

### 2.3 Acid digestion and elemental analysis

After harvest, the roots and nodules were washed and ultra-sonicated with CaCl_2_ (0.01 M) for 5 min followed by a further 3 min of ultra-sonication with HQ-water. The above-ground biomass was rinsed with deionized water and patted dry. The roots, shoots, leaves, and nodules were separated and dried at 60°C for 1 week and subsequently ground to a homogenous fine powder using a Retsch mill (Retsch MM20). Then, approximately 200 mg of dry, ground plant material was digested in a matrix containing 5 mL HNO_3_, 1 mL H_2_O_2_, and one drop of 1-octanol. For biomass less than 100 mg, the amount of acid and peroxide was reduced by 50%. The digestion was performed using an open digestion unit (Velp Scientifica). The volume of digests was made up to 50 mL/25 mL with HQ-water resulting in ∼6.5% HNO_3_. The nutrients and tungsten concentrations in the digests were analyzed using inductively coupled plasma optical emission spectroscopy (ICP-OES Optima 3000 XL Perkin Elmer). The translocation factors (TF) for individual nutrient and tungsten were calculated by dividing the tissue concentrations of shoots by the concentrations in the root.

### 2.4 Sequential extraction of metabolites and proteins

Frozen plant material was ground to a fine powder on LN_2_ using a porcelain mortar. A total of 60 mg (fresh weight) was then used for metabolite and protein extraction for spectrophotometric and mass spectrometric analyses using an adapted sequential extraction procedure by Laware (2015). The extraction procedure for photosynthetic pigments and primary and secondary metabolites is briefly described below. A detailed description of the extraction procedure as well as the protocols for integrative estimation of key plant metabolites can be found in the **Supplements (Supplementary S1_1)**.

#### 2.4.1 Extraction of photosynthetic pigments and lipids

For the extraction of photosynthetic pigments and lipids, the samples were covered with 1 mL of pre-chilled 80% acetone, subsequently vortexed, and centrifuged at 10,000 × *g* for 10 min. The supernatant was collected in a new tube, while the pellet was re-extracted two times with 0.5 mL of 80% acetone and centrifugation at 10,000 × *g* for 10 min. After pooling the supernatants, 200 µL was transferred to a new tube, and the volume was made up to 1 mL with 80% acetone. The absorbance of diluted samples (1:5) was measured at 663, 646, and 470 nm with a spectrophotometer for the estimation of pigments according to Lichtenthaler and Wellburn (1983).

#### 2.4.2 Extraction of primary and secondary metabolites

The pellet obtained from the acetone extraction was extracted in 1 mL of 80% methanol, vortexed, and incubated at 95°C for 30 min. After this, the samples were centrifuged at 20,000 × *g* for 10 min and the supernatant was collected in a new tube. The pellet was then re-extracted in 1 mL 80% ethanol, incubated at 95°C for 30 min, and subsequently cooled on ice and centrifuged at 20,000 × *g* for 10 min. The supernatant was pooled with the supernatant from the previous methanol extraction. The pellet was used for the extraction of soluble proteins.

For further metabolite analysis, two aliquots per sample were prepared by pooling 1 mL of the acetone extract and 1 mL of the combined ethanol–methanol extracts. One aliquot was used to immediately perform the photometric metabolite marker assays. The second aliquot was used for the GC–MS analysis. Therefore, 5 µL of 1 mM phenyl- β-D-glucopyranoside (PGP) and 10 mM pentaerythritol (PE), respectively, were added to each sample as internal standards, and the extracts were dried under nitrogen flow and stored at -80°C until measurement. The QCs and blanks were prepared accordingly.

#### 2.4.3 GC–MS analysis

The dried samples, QCs, and blanks were redissolved in 20 μL of 40 g L^−1^ methoxyamine hydrochloride in pyridine and incubated at 30°C for 90 min. Then, 80 μL N-methyl-N-trimethylsilyl-trifluoroacetamide (MSTFA) was added, and the samples were incubated at 37°C for another 30 min before they were centrifuged for 2 min at 14,000 × *g*. Finally, 70 μL of the supernatant was transferred to a MS vial, and caps with septum were applied.

The GC–MS measurement of samples, QCs, and blanks was performed with an Agilent 7890B GC coupled to a LECO Pegasus^®^ BT GC-TOFMS (LECO Corporation, MI, USA). The measurements were performed in split 100 and split 5 mode. If the concentrations of analytes were too high and resulted in detector overload, split 100 was used for peak integration. As RI marker, alkane analytical standards (C10–C40) (Sigma-Aldrich) were used and injected separately at the beginning and end of each batch. The front inlet temperature was set to 230 for the entire run, and the initial oven temperature was set to 70°C for 1 min and then increased to an end temperature of 330°C with a rate of 9°C min^−1^. The target temperature was kept for 8 min. The data acquisition rate was set to 10 spectra per second with a detector voltage of 1,550 V and acquisition delay of 5 min. The mass range was set from 50 to 600 *m*/*z*, and the extraction frequency was set to 30 kHz. Raw data was processed with the LECO Chroma-TOF^®^ software (LECO^®^ Corporation, MI, USA). The GC-metabolomic data used for the statistical analysis can be found in the supplement (**Supplementary Table S8**).

#### 2.4.4 Photometric measurement of sugars, secondary metabolites, and antioxidant capacity

The estimation of soluble carbohydrates was performed according to Hansen and Møller (1975). The assays for the determination of total phenols and flavonoids were performed according to Chang et al. (2002) and Rebaya et al. (2015). Condensed tannin contents were assayed according to Broadhurst and Jones (1978). Antioxidant capacity [2,2-diphenyl-1-picrylhydrazyl (DPPH) radical-scavenging activity] was determined according to Rebaya et al. (2015). The percentage of inhibition of oxidation was calculated from obtained absorbance at 517 nm by the equation: % inhibition = [(Abs control - Abs test)/Abs control] × 100. Subsequently, the half-maximal inhibitory concentration (IC50) for each sample was calculated by plotting the dilution curves against % inhibition. IC50 represents the amount of sample (µg FW/mL) needed to reduce the initial DPPH absorbance by 50%. The lower the IC50, the higher the antioxidative activity.

#### 2.4.5 Protein extraction

The pellet obtained from the acetone, ethanol, and methanol extraction was air-dried on ice and subsequently resuspended in 1 mL of protein extraction buffer [urea 8 M; 4-(2-hydroxyethyl)-1-piperazineethanesulfonic acid (HEPES) 50 mM, pH 7.8], incubated at 4°C for 60 min, and later centrifuged (10,000 × *g*, 10 min, 4°C). The supernatant was transferred into a 15-mL falcon tube, covered with six volumes of ice-cold acetone with 0.5% beta-mercaptoethanol, and the proteins were precipitated at -20°C overnight. This was followed by 10 min of centrifugation at 4°C at 4,000 × *g*. The pellet was washed three times with 1 mL ice-cold acetone and then air-dried under the hood at room temperature for 10 min. The pellet was re-dissolved in buffer containing 8 M urea and 50 mM HEPES at pH 7.8 for protein content determination using a Quick Start Bradford protein assay from Bio-Rad Laboratories and a Perkin Elmer photometer (EnSpire 2300 Multilable Reader). Protein content was determined using a standard curve of Quick Start Bovine Serum Albumin standards, also obtained from Bio-Rad.

##### 2.4.5.1 Protein reduction and alkylation

Protein concentration was normalized to 10 μg protein in 8-M-urea buffer with 5 mM dithiothreitol (DTT) and incubated for 45 min at 37°C and 700 rpm. Then, indole-3-acetic acid (IAA) was added to a concentration of 10 mM, and the samples were incubated for another 60 min at 23°C and 700 rpm. Alkylation was stopped by adjusting the DTT concentration in the sample to 10 mM. The samples were subsequently put at 23°C for another 15 min at 700 rpm.

##### 2.4.5.2 Protein digestion

The urea sample concentration was diluted to 4 M by adding an equal volume of 100 mM ammonium bicarbonate (AmBic) in 20% acetonitrile (ACN). Subsequently, 0.1 μg Lys-C was added, and the samples were incubated at 30°C for 5 h at 500 rpm in the dark (Niessen et al., 2006).

Thereafter, another volume of 10% acetonitrile containing 25 mM AmBic, 10 mM CaCl_2_, and 5 mM DTT was added to the sample, reducing the final urea concentration from 4 M to 2 M. Moreover, 3 μL of trypsin beads (Poroszyme, Applied Biosystems) were added, and the samples were incubated for 14 h at 37°C at 10 rpm in a rotating hybridization oven (Boekel little shot III). After centrifugation at 10,000 × *g* at 4°C, the digested proteins were desalted with Agilent Bond Elut OMIX C18 pipette-based SPE stage tips from Agilent Technologies, according to the manufacturer’s instructions, dried in a vacuum concentrator (ScanSpeed MaxiVac), and stored at -80°C.

##### 2.4.5.3 ESI LC–MS/MS measurement

The dried protein digests were re-dissolved in 100 μL 2% acetonitrile (ACN) and 0.1% formic acid (FA), ultra-sonicated for 15 s, and subsequently centrifuged at 4°C for 10 min at 21,000 × *g*. For the separation of peptides, a uHPLC system (Dionex Ultimate 3000) with a flow rate of 300 μL min^-1^ with a Thermo scientific Easy Spray column was used. Furthermore, 1 μg protein was loaded onto the column and eluted with a 145-min gradient from 2% to 90% ACN 0.1% FA. MS analysis was carried out using a Thermo scientific velos rvo ion trap and Thermo scientific LTQ Orbitrap Elite with 1 MS1 scan (FTMS) with a scan range of 350–1,800 *m*/*z* and 20 MS2 scans (ITMS) of the most abundant *m*/*z* ratios acquired from MS1. A twofold charge was set as the default charge state; the unassigned charge states as well as +1 charge states were rejected. The size of the exclusion mass list was set to 500 (with a duration of 60 s), and the exclusion mass width was set to 5 ppm with one repeated count of 30 s; the minimal required signal was set to 10,000.

##### 2.4.5.4 Protein identification and quantification

Analysis of mass spectral data was performed using MaxQuant (2.0.3.1) as described in Preiner et al. (2019). Briefly, raw files were searched against a combined FASTA file for *Glycine max* and *Bradyrhizobum japonicum* with 115,028 entries (http://www.uniprot.org/25.04.2019). A maximum of two missed cleavages as well as a maximum of three modifications were allowed per peptide (oxidation, N-terminal acetylation), and tolerance for precursor mass was set to 4.5 ppm (FTMS) and 0.6 Da (ITMS). Data was additionally searched against a database of revert sequences in a target–decoy approach to eliminate matching by chance. Only high-confidence peptides [false discovery rate (FDR) <0.01%] as well as proteins with at least two distinct identified peptides passed the criteria for identification. Additionally, the FDR-based “matching between runs” algorithm was used (Cox and Mann, 2008). For relative quantification, LFQ intensities were used.

Proteins were functionally categorized based on sequence similarity with proteins of other organisms via BLAST and RPS-BLAST against reference databases using the Mercator 3.6 sequence annotation tool (http://plabipd.de/portal/mercator-sequence-annotation) (Lohse et al., 2014). Proteins that could not be assigned to specific functional categories were later manually assigned according to protein name and function with Uniprot or remained “not assigned”. The proteomic data has been deposited to the ProteomeXchange Consortium with the dataset identifier PXD047532 via the CCMS MassIVE data repository and can be downloaded with the MassIVE identifier MSV000093570 (doi:10.25345/C5ZC7S52C).

#### 2.4.6 Estimation of starch concentration

The pellets obtained after protein extraction were washed two times with 1 mL 80% ethanol to remove remaining urea and sugars and subsequently dried at room temperature for approximately 10 min to let the residual solvents evaporate. The following starch assay was performed according to Nägele and Heyer (2013) as described in Preiner et al. (2019).

### 2.5 Statistical analysis

For statistical analysis, data was only used if values were present in three or more replicates of at least one treatment. If less than half of the values were missing in one treatment, the missing values were imputed by the k-nearest neighbor algorithm; if more values were missing, the values were imputed with the smallest value detected throughout all samples divided by two. Two samples of one treatment (tungsten N fix root/leaf) were lost during sample processing (metabolite extraction) and thus were not imputed as they were considered systemically missing (*n* = 3 to 4). The statistical analysis of data from the sequential extraction protocol, starch assay, GC–MS, and ICP-OES as well as the physiological data was performed using Infostat software (InfoStat, RRID : SCR_014310) (Di Rienzo et al., 2012). Data was analyzed by using a linear mixed model and DGC *post hoc* test. If necessary, heteroscedasticity was corrected by selecting the varIdent model in Infostat software.

For the statistical analysis of the protein data, an analysis of variance (ANOVA) followed by a Tukey HSD *post hoc* test was performed using R-studio. The LFQ intensities of each treatment were averaged, and the ratios between control and high tungsten treatments were calculated. Proteins were considered significantly changed when the change (ratio) between the compared treatments was higher or at least twofold as well as with a *p*-value below 0.05. *P*-value correction was performed using Benjamini–Hochberg to account for false positives due to multiple testing. Adjusted P-values are provided in the summary tables in the **Supplement** (**Supplementary Tables S5**, **S6**).

Additionally, a principal component analysis (PCA) was performed with R-studio to filter for the effect size of individual variables. For the principal component analysis, a new data matrix was created for roots as well as leaves. Each matrix contained all metabolites and significantly changed proteins that were detected in at least three out of four replicates of at least one treatment. The PCA was performed using prcomp-package (principal component analysis, RRID : SCR_014676) using log^2^-transformed data. Heat maps were created using the R package gplots. Euclidian distance with complete linkage was used for clustering. The PCA loadings for proteins can be found in **Supplementary Tables S5**, **S6**. The loadings for metabolites are provided in **Supplementary Tables S3**, **S4**. GO enrichment analysis as well as annotation for molecular function and molecular process was performed with the Panther 18.0 analysis tool using the PANTHER overrepresentation test (Released 20231017) and GO Ontology database (DOI: 10.5281/zenodo.8436609, released 2023-10-09) for *Glycine max* (Ashburner et al., 2000; Thomas et al., 2022; Aleksander et al., 2023). As statistical test, Fisher’s exact was chosen, and false discovery rate (FDR) was calculated for significant enrichments (**Supplementary Table S7**).

## 3 Results

### 3.1 Tungsten exposure hampers plant growth and photosynthesis

#### 3.1.1 Biomass and protein concentrations

**Figure 1:**
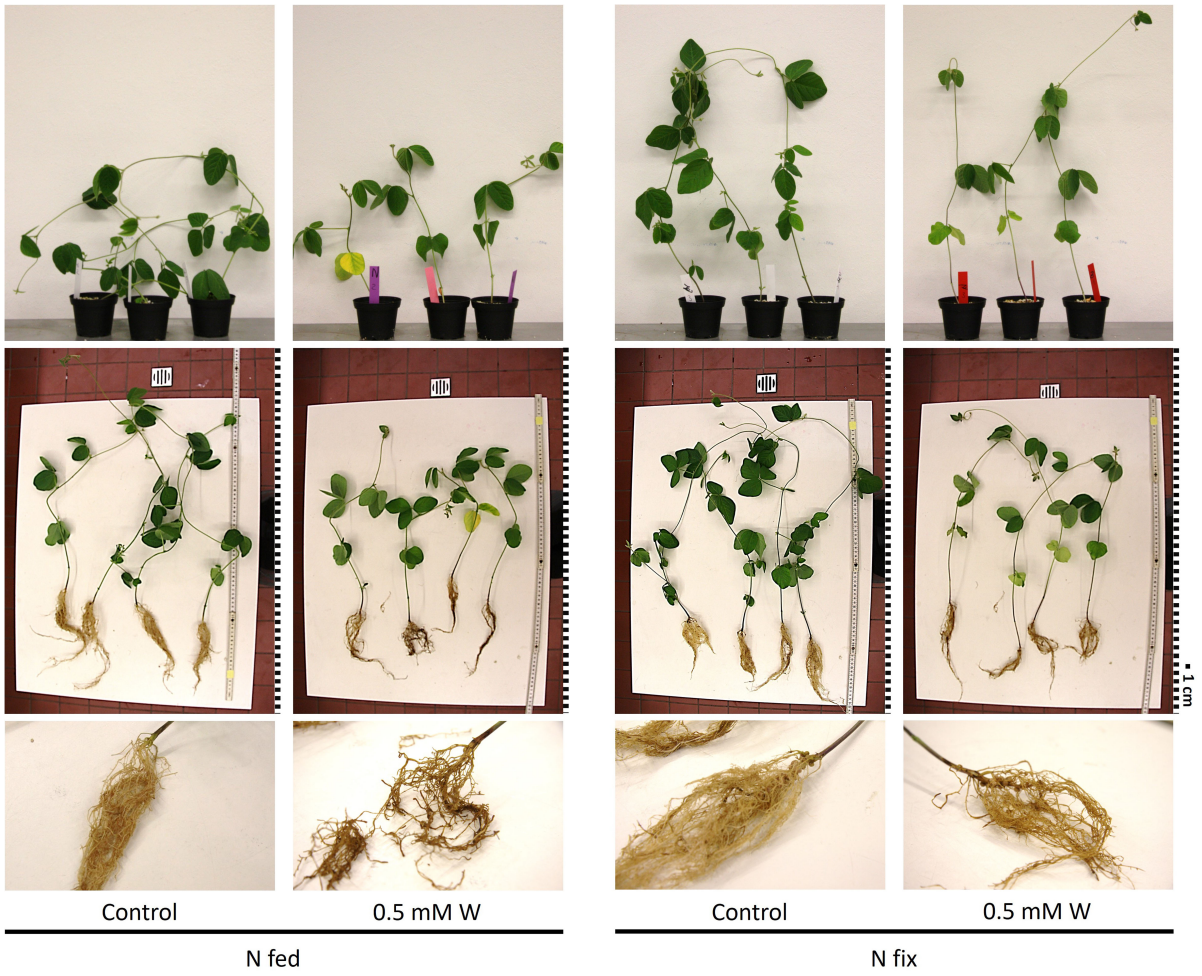
Pictures of soybean plants (*Glycine max* cv Primus) grown semi-hydroponically without (control) or with tungsten (0.5 mM W) and two different nitrogen supply regimes (N fix: inoculated with *B. japonicum*, weeks 1 and 2: 0.25 mM KNO_3_ and weeks 3–5: zero N; N fed: weeks 1–5: 10 mM KNO_3_). Brightness was increased by +40% in all pictures; the pictures of whole plants in the table were also sharpened by +50%. For better comparability, the pictures were resized and a graphical representation of the scale was added. Each square represents 1 cm.

The effects of tungsten on shoot and root biomass of N_2_ fixing (N fix) and KNO_3_-fertilized (N fed) plants are presented in **Table 1**. Overall, the effects of tungsten on plant growth were clearly visible (**Figure 1**). While tungsten-stressed N fed plants exhibited robust, stunted stems, N fix plants had smaller leaves and thinner stems but did not show reduced shoot growth (length in centimeters). This is also reflected in biomass data, as shoot and root biomass (mg DW), respectively, were significantly reduced in both N regimes (**Table 1**). Although root length was not affected in either N regime, the roots of N fed plants showed morphological changes such as blackened tips and a coral-like morphology (**Figure 1**). Nodule count was not affected by the presence of tungsten. Although the seeds were sterilized, individual nodules were found on the roots of N fed controls (**Table 1B**). These nodules did not show the typical red coloration when cut in half and are regarded as artifacts.

**Table 1:** Shoot (**A**) and root (**B**) length (cm), fresh weight (g), dry weight (mg), nodule count as well as total soluble protein concentrations (μg mg^-1^ fwt.) of soybean (*Glycine max* cv Primus) grown semi-hydroponically without (control) or with tungsten (0.5 mM W) and two different nitrogen supply regimes (N fix: inoculated with *B. japonicum*, weeks 1 and 2: 0.25 mM KNO_3_ and weeks 3–5: zero N; N fed: weeks 1–5: 10 mM KNO_3_).

		N fed								N fix							
		Control				0.5 mM W				Control				0.5 mM W			
	Unit	Means		S.E.	sig.	Means		S.E.	sig.	Means		S.E.	sig.	Means		S.E.	sig.
**(A) SHOOT**																	
**Length**	cm	68.3	±	6.07	**A**	41.3	±	5.12	**B**	67.3	±	7.27	**A**	54.8	±	6.14	**A**
**Fresh Weight**	g	4.76	±	0.11	**A**	3.24	±	0.28	**B**	4.52	±	0.24	**A**	2.19	±	0.18	**C**
**Dry Weight**	mg	1.27	±	0.06	**A**	0.98	±	0.10	**B**	1.29	±	0.09	**A**	0.69	±	0.03	**C**
**Protein Content Leaf**	μg mg^-1^ fwt.	116	±	13.1	**A**	138	±	30.7	**A**	98.4	±	4.09	**A**	131	±	23.6	**A**
**(B) ROOT**																	
**Length**	cm	26.8	±	2.29	**A**	24.0	±	1.08	**A**	28.0	±	3.19	**A**	22.3	±	2.87	**A**
**Fresh Weight**	g	2.97	±	0.18	**A**	1.61	±	0.23	**B**	2.68	±	0.06	**A**	1.33	±	0.11	**B**
**Dry Weight**	mg	0.25	±	0.02	**A**	0.18	±	0.02	**B**	0.32	±	0.03	**A**	0.18	±	0.02	**B**
**Nodule Count**	pcs	2.50	±	1.99	**B**	0.50	±	1.99	**B**	21.0	±	3.49	**A**	19.8	±	1.11	**A**
**Protein Content Root**	μg mg^-1^ fwt.	12.1	±	3.29	**A**	6.39	±	1.74	**A**	6.80	±	0.29	**A**	8.05	±	1.09	**A**

Values are means ± S.E. (biomass n = 4, protein and pigment content n=3-4). Letters indicate significant differences between the different tungsten treatments and nitrogen regimes (ANOVA, post hoc DGC, p < 0.05).

Although not significant, protein content in leaves was elevated in tungsten-treated plants compared to the controls (**Table 1A**). In N fed roots, on the contrary, protein content was significantly reduced compared to the N fed controls and roots of N fix plants (**Table 1B**).

#### 3.1.2 Photosynthetic pigments, phenolic compounds, and antioxidant capacity

**Figure 2:**
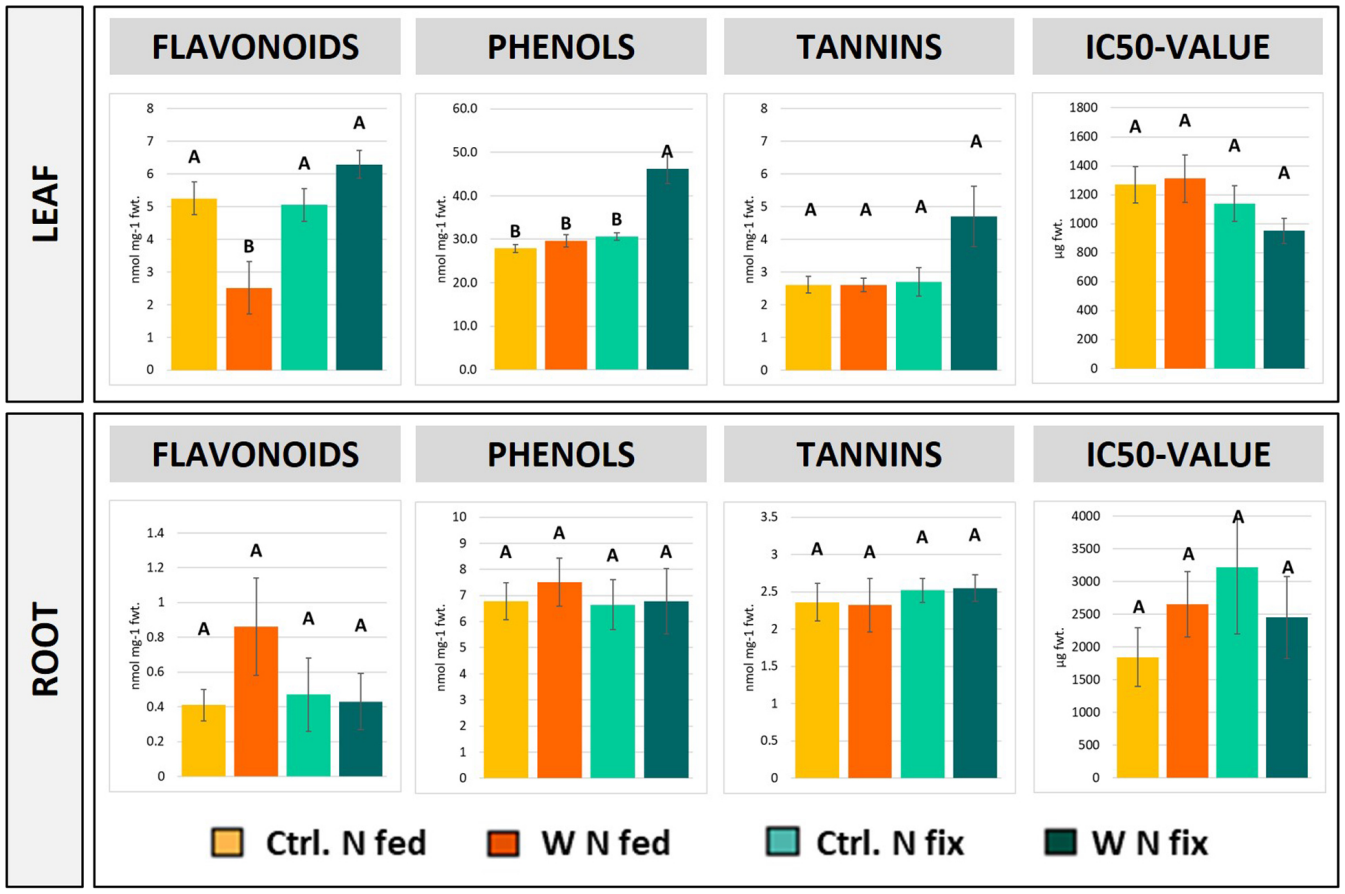
Concentrations of total flavonoids, phenolic compounds, tannins as well as IC50 values in the leaf and root tissue of soybean (*Glycine max* cv Primus) grown semi-hydroponically without (Ctrl.) or with 0.5 mM tungsten (W) and two different nitrogen supply regimes (N fix: inoculated with *B. japonicum*, weeks 1 and 2: 0.25 mM KNO_3_ and weeks 3–5: zero N; N fed: weeks 1–5: 10 mM KNO_3_). The values are means ± SE (*n* = 3 to 4). The letters indicate significant differences between the different tungsten treatments and nitrogen regimes (ANOVA, *post hoc* DGC, *p* < 0.05).

The concentrations of total phenolic compounds were significantly increased in the leaves of tungsten-stressed N fix plants compared to N fed plants and both control treatments (**Figure 2**). Additionally, the concentrations of total flavonoids and tannins were elevated (not significant) in tungsten-stressed N fix plants compared to the control. The tungsten-stressed N fed plants exhibited significantly decreased flavonoid concentrations in leaves compared to the control (**Figure 2**). The concentrations of total phenolic compounds (total phenols, total flavonoids, and total tannins) were not affected in the roots of tungsten-treated plants (**Figure 2**). Although not significant, the tungsten-stressed N fix plants showed consistently lower IC50 values compared to the control. Conversely, the tungsten-stressed N fed plants showed higher IC50 values, indicating lower antioxidative capacity (**Figure 2**). Photosynthetic pigments are shown in **Supplementary Table S1A**. Tungsten exposure did not affect the concentrations of photosynthetic pigments in our experimental setup. No significant difference between the treatments could be observed in chlorophyll a and b and carotenoid contents.

#### 3.1.3 PAM measurements

The results of chlorophyll fluorescence measurements are shown in the supplement (**Supplementary Figure S1**; **Supplementary Table S1B**). The tungsten-stressed N fix and N fed plants showed a significantly lower effective photochemical quantum yield [Y(II)] across all PAR intensities as well as photochemical fluorescence quenching coefficient (qP) at PAR 634 compared to their respective controls (**Supplementary Figures S1A, B**; **Supplementary Table S1B**). However, no difference in maximum photochemical quantum yield (Fv/Fm) was found between controls and tungsten-treated plants (**Supplementary Table S1B**). Furthermore, the levels of non-photochemical quenching (qN/NPQ) were lowest in control N fix plants and significantly elevated in the tungsten treatments as well as in nitrogen-fertilized control plants (**Supplementary Figure S1C**). The relative electron transport rate (ETR) was highest in N fix control, followed by N fed control (**Supplementary Figure S2D**).

### 3.2 Reduced root-to-shoot translocation of tungsten in N fix plants

#### 3.2.1 Tungsten concentrations

The tungsten concentrations in roots were significantly higher in N fix plants compared to N fed plants (**Table 2**). Conversely, N fed plants showed higher levels of tungsten in stems and leaves compared to their N fix counterparts. However, this was not statistically significant. These differences in tungsten tissue distribution result in a significantly higher root-to-shoot translocation factor for tungsten in N fed plants [1.18 (N fed 0.5mM W)] compared to N fix plants [0.57 (N fix 0.5mM W)] (**Table 2**).

**Table 2:** Tungsten concentrations in leaves, stems and roots, as well as root to shoot translocation factor of soybean (*Glycine max* cv Primus) grown semi-hydroponically without (Control) or with tungsten (0.5 mM W) and two different nitrogen supply regimes (N fix: inoculated with *B. japonicum*, weeks 1 and 2: 0.25 mM KNO_3_ and weeks 3–5: zero N; N fed: weeks 1–5: 10 mM KNO_3_).

		N fed								N fix							
		Control				0.5 mM Tungsten				Control				0.5 mM Tungsten			
Plant organ	Unit	Means		S.E.	sig.	Means		S.E.	sig.	Means		S.E.	sig.	Means		S.E.	sig.
**Leaves**	mg kg^-1^	LOQ				286.2	±	44.5	**A**	LOQ				218.2	±	20.1	**A**
**Stems**	mg kg^-1^	LOQ				270.7	±	73.5	**A**	LOQ				150.2	±	14.9	**A**
**Roots**	mg kg^-1^	LOQ				473.7	±	30.7	**B**	LOQ				701.8	±	92.1	**A**
**Translocation Factor (TF)**		LOQ				1.18	±	0.21	**A**	LOQ				0.57	±	0.14	**B**

Values represent means ± S.E. (n = 4). Letters indicate significant differences between the different tungsten treatments and nitrogen regimes (ANOVA, post hoc DGC, p < 0.05). LOQ, limit of quantification.

#### 3.2.2 Macro- and micronutrients

The concentrations of macro- and micronutrients in roots, stems, and leaves are shown in **Supplementary Table S2**. In the leaves and stems of tungsten-stressed N fix plants, the K, Mn, and Zn concentrations were significantly reduced (**Supplementary Table S2A**). In stems, additionally, the Ca and Mg levels were significantly lower compared to the control (**Supplementary Table S2B**). Conversely, tungsten exposure led to a significant increase of S and Fe (in leaves) as well as a decrease of Mn concentrations in the stems and leaves of N fed plants. The roots of N fix plants exhibited significantly lower levels of Mn, K, Mg, and S; in the roots of N fed plants, K, Mg, and S were significantly decreased (**Supplementary Table S2C**). Additionally, both N regimes showed elevated levels of Fe throughout all organs.

While the translocation (**Supplementary Table S2D**) of Mg, K, S, and Mn was significantly higher in N fix plants compared to the control, there was a significantly lower translocation of Zn and Ca. In N fed plant, K, Mg, and S showed a significantly higher translocation factor and P, Cu, and Zn a significantly lower translocation factor.

### 3.3 Multivariate statistics

**Figure 3:**
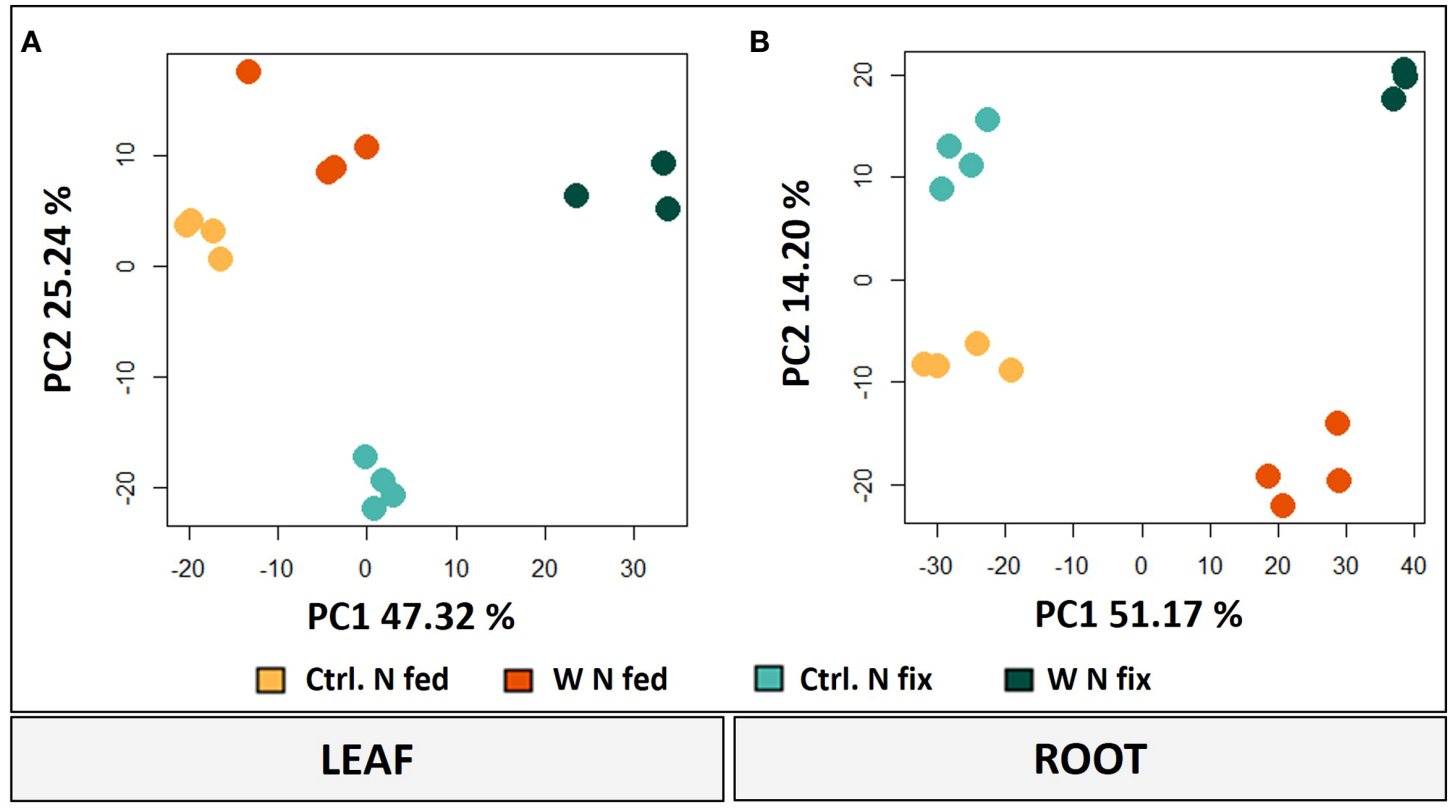
PCA plots (PC1/PC2) of leaves **(A)** and roots **(B)** including log2-transformed metabolite concentrations and LFQ intensities of significantly changed proteins (*n* = 3 to 4, ANOVA, *post hoc* Tukey, *p* < 0.05). Soybean plants (*Glycine max* cv Primus) were grown semi-hydroponically without (Ctrl.) or with 0.5 mM tungsten (W) in nutrient solution and two different nitrogen supply regimes (N fix: inoculated with *B. japonicum*, weeks 1 and 2: 0.25 mM KNO_3_ and weeks 3–5: zero N; N fed: weeks 1–5: 10 mM KNO_3_).

The principal component analysis (PCA) of both leaves and roots shows a clear separation between the different treatments. PC1 to PC2 contribute most to the difference between the treatments and thus were considered for further analysis (**Figure 3**). In leaves, PC1 accounts for 47.32% and PC2 for 25.24% of the difference between the treatments (**Figure 3A**). While PC1 represents the difference between controls and tungsten-treated plants, PC2 represents the difference between the N fix control and the nitrogen-fertilized plants. Furthermore, tungsten-stressed N fix plants and controls are separated on PC2.

In roots, PC1 is responsible for 51.17% of the variance and clearly separates the tungsten-stressed plants and controls (**Figure 3B**). PC2 explains another 14.20% of the variance and shows the difference between the two N regimes. To identify the proteins and metabolites responsible for the separation, a threshold with the largest loadings was set at ±0.065 (**Supplementary Figure S2**).

#### 3.3.1 Leaf metabolites

**Figure 4:**
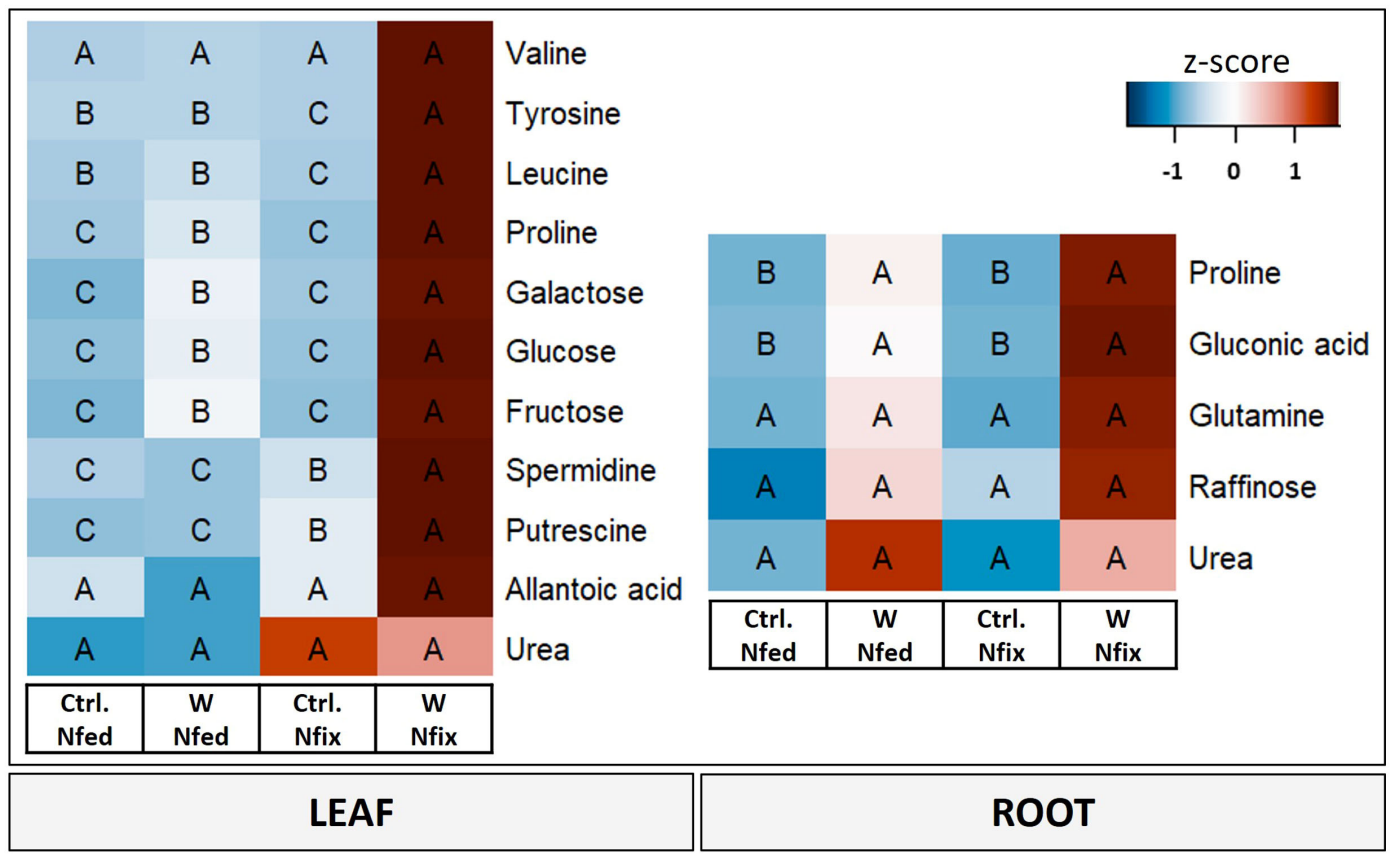
Heat map of leaf and root metabolites with PC1 loadings higher than ±0.65. Soybean plants (*Glycine max* cv Primus) were grown semi-hydroponically without (Ctrl.) or with 0.5 mM tungsten (W) in the nutrient solution and two different nitrogen supply regimes (N fix: inoculated with *B. japonicum*, weeks 1 and 2: 0.25 mM KNO_3_ and weeks 3–5: zero N; N fed: weeks 1–5: 10 mM KNO_3_). The colors represent z-transformed mean values (*n* = 3 to 4). The letters indicate significant differences between the different tungsten treatments and nitrogen regimes (ANOVA, *post hoc* DGC, *p* < 0.05).

Principal component analysis revealed that amino acids (valine, tyrosine, leucine, and proline), polyamines (spermidine and putrescine), carbohydrates (glucose, fructose, and galactose) and nitrogen compounds (urea and allantoic acid) (**Figure 4**) were the metabolites with the highest loadings ( ± 0.065) and responsible for the separation on PC1 (i.e., tungsten treatments). Detailed results for individual metabolites as well as PCA loadings can be found in the supplements (**Supplementary Table S3**). Overall, the tungsten-treated plants showed higher leaf tissue concentrations of soluble sugars compared to the control treatments (**Supplementary Table S3A**). Additionally, the levels of total amino acids were significantly higher in N fix plants (**Supplementary Table S3B**) and significantly lower in N fed plants compared to their respective control. Changes in metabolite levels were more strongly pronounced (higher fold change) in N fix plants (**Supplementary Table S3**).

PC2, which separates N fix controls from all the other treatments, is again determined by amino acids (leucine, isoleucine, proline, valine, tyrosine, phenylalanine, asparagine, and alanine), sugars and sugar alcohols (galactose, glucose, fructose, raffinose, and alloinositol), nitrogen compounds (urea), and the terpenoid lupeol (**Supplementary Figure S3**).

#### 3.3.2 Root metabolites

Proline, gluconic acid, glutamine, and raffinose as well as urea were the metabolites with the highest loadings ( ± 0.065) in PC1 and responsible for the separation between the controls and the tungsten-stressed plants (**Figure 4**). Overall, changes of metabolite levels were less pronounced in roots (**Supplementary Table S4**). The difference between the N regimes (PC2) is determined by carbohydrates (glucose and raffinose), nitrogen compounds (allantoic acid), polyamines (putrescine and spermidine), and malonic acid (**Supplementary Figure S3**).

#### 3.3.3 Leaf proteome

Of 2,515 proteins that were identified in leaves, 1,669 were detected in at least three out of four replicates of at least one treatment. Of these proteins, 233 proteins showed a significantly changed abundance between the tungsten-treated plants and the controls (SI). A vast majority (195) of these proteins were changed in N fix leaves. Only 38 proteins showed altered concentrations in N fed leaves, of which 14 were also changed in the leaves of N_2_-fixing plants (**Supplementary Figure S4**). A full list of significantly changed leaf proteins can be found in the supplements (**Supplementary Table S5**).

**Figure 5:**
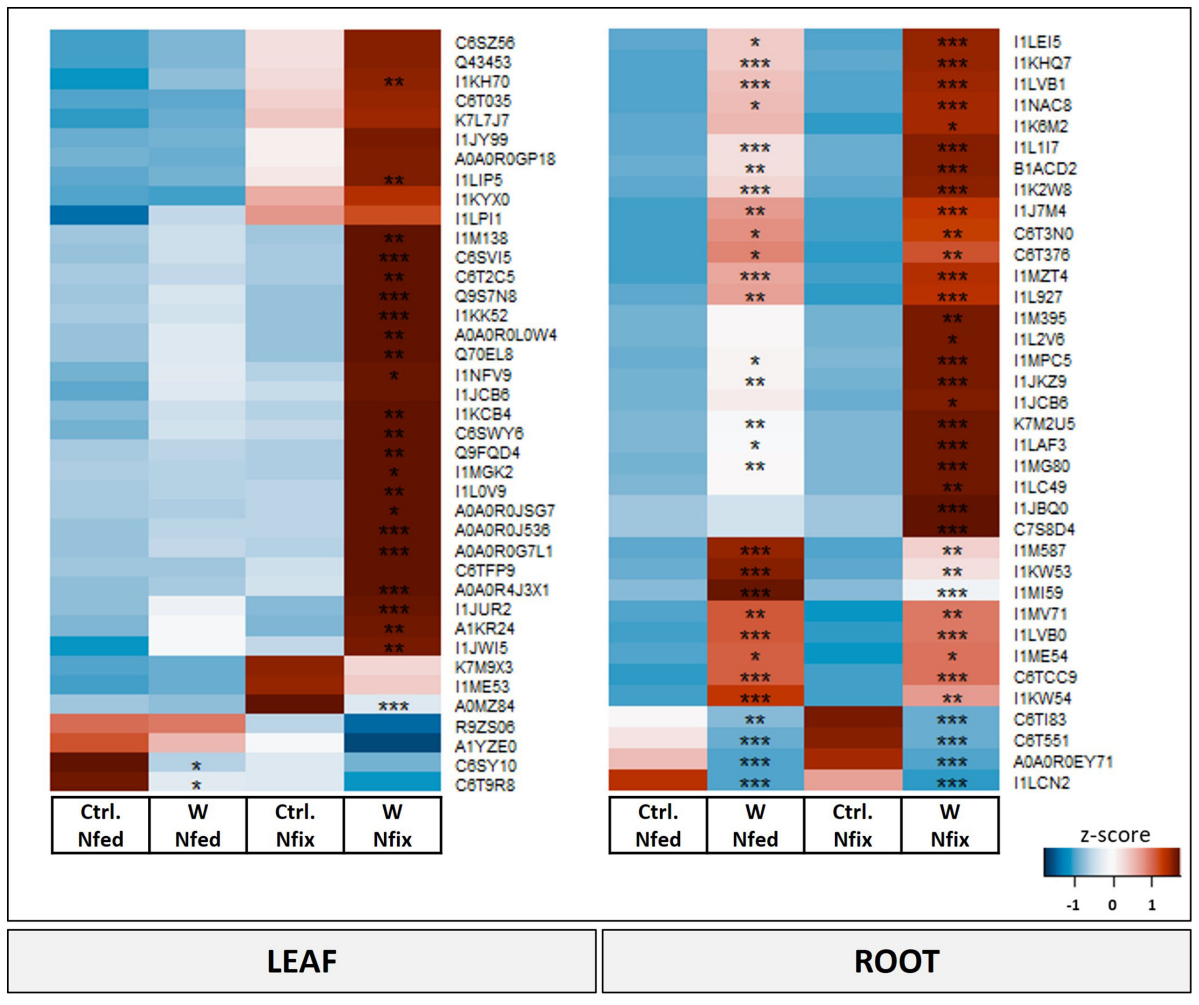
Heat map of significantly changed leaf and root proteins (ANOVA, *post hoc* Tukey, *p* < 0.05) with PC1 loadings higher than ±0.65. Soybean plants (*Glycine max* cv Primus) were grown semi-hydroponically without (Ctrl.) or with 0.5 mM tungsten (W) in the nutrient solution and two different nitrogen supply regimes (N fix: inoculated with *B. japonicum*, weeks 1 and 2: 0.25 mM KNO_3_ and weeks 3–5: zero N; N fed: weeks 1–5: 10 mM KNO_3_). The colors represent z-transformed mean values (*n* = 3 to 4). Significant difference is indicated with an asterisk (ANOVA, *post hoc* Tukey, *p* < 0.05).

Proteins with the highest loadings are shown in **Figure 5**. A detailed list with names and *p*-values of proteins shown in **Figure 5** can be found in **Table 3**. The functional categories of proteins with the highest positive loadings on PC1 (**Figure 5**) are “external stimuli response” (e.g., annexin D1, dehydrin, endochitinases, and Bet v I/major latex protein domain-containing protein), “protein biosynthesis” (50S ribosomal protein L12 and chloroplastic), “protein degradation” (pepsin-type protease), “protease/enzyme inhibition” [protease/α-amylase inhibitor (AAI) and vacuolar inhibitor of fructosidase 1], “lipid metabolism” (GDSL esterase/lipase, non-specific lipid transfer protein), “cell wall”, “enzyme classification” (lipoxygenases), “carbohydrate metabolism” [granule-bound starch (amylose) synthase], “protein modification” (glutathione-S-transferase), “redox homeostasis” (copper/zinc superoxide dismutase, and peroxidases), “nutrient uptake” (non-haem-ferritin and calcium ion binding), and “not assigned” (**Table 3A**). Proteins (**Figure 5**; **Table 3A**) with the largest negative loadings showed a decreased abundance (ratio higher than 1) in tungsten-treated plants and can be assigned to the functional categories “photosynthesis” (e.g., glyceraldehyde 3-phosphate dehydrogenase and a large subunit of ribulose-1,5-bisphosphat carboxylase/oxygenase), “phytohormone” (GASA precursor polypeptide), and “carbohydrate metabolism” (granule-bound starch synthase). The GO enrichment analysis revealed GO biological processes “response to stress (GO:0006950)”, “response to stimulus (GO:0050896)”, “reactive oxygen species metabolic process (GO:0072593)”, “response to toxic substance (GO:0009636)”, and “defense response (GO:0006952)” as well as their subcategories to be significantly enriched compared to the reference list (**Table 3A**; **Supplementary Table S7A**).

**Table 3:** Significantly changed leaf (A) and root (B) proteins with PC1 loadings higher than ±0.65.

					N fed		N fix	
					Ctrl. vs. W		Ctrl. vs. W	
Uniprot ID	Annotation (UniprotKB/Uniref100)	Mercator categories	GO terms (biological process)	sig. E.A.	ratio	p-val.	ratio	p-val.
**(A) Leaf Proteins PC1 cutoff <0.65**		** **	** **	** **	** **	** **	** **	** **
**C6SZ56**	copper/zinc superoxide dismutase	Redox homeostasis	n.a.	** **	0.41	0.99	0.51	0.36
**Q43453**	Stress-induced protein SAM22	External stimuli response	defense response (GO:0006952)	**x**	0.53	0.99	0.53	0.34
**I1KH70**	Lipoxygenase	Enzyme classification	lipid metabolic process (GO:0006629)	** **	0.39	0.46	0.55	0.00
**C6T035**	Uncharacterized protein	n.a.	n.a.	** **	0.80	1.00	0.58	0.15
**K7L7J7**	Lipoxygenase	Enzyme classification	lipid metabolic process (GO:0006629)	** **	0.66	0.99	0.62	0.19
**I1JY99**	Peroxidase	Redox homeostasis	response to oxidative stress (GO:0006979)	**x**	0.66	1.00	0.42	0.12
**A0A0R0GP18**	Uncharacterized protein	n.a.	electron transport chain (GO:0022900)	** **	1.20	1.00	0.46	0.09
**I1LIP5**	EF-hand domain-containing protein	Nutrient uptake		** **	0.87	1.00	0.50	0.01
**I1KYX0**	Uncharacterized protein	protease/enzyme inhibitor	n.a.	** **	1.13	1.00	0.69	0.42
**I1LPI1**	50S ribosomal protein L12, chloroplastic	Protein biosynthesis	translation (GO:0006412)	** **	0.23	0.51	0.85	0.92
**I1M138**	Aspartic-type endopeptidase activity	Protein degradation	proteolysis (GO:0006508)	** **	0.43	0.96	0.07	0.01
**C6SVI5**	Uncharacterized protein	n.a.	n.a.	** **	0.43	0.84	0.11	0.00
**C6T2C5**	Uncharacterized protein	protease/enzyme inhibitor	n.a.	** **	0.40	0.99	0.04	0.00
**Q9S7N8**	Seed maturation protein PM21	n.a.	n.a.	** **	0.25	0.68	0.03	0.00
**I1KK52**	α-amylase inhibitor (AAI)	protease/enzyme inhibitor	systemic acquired resistance (GO:0009627)		0.26	0.72	0.01	0.00
**A0A0R0L0W4**	Peptidase A1 domain-containing protein	Protein degradation	proteolysis (GO:0006508)	** **	0.27	0.89	0.05	0.01
**Q70EL8**	Dehydrin	External stimuli response	n.a.	** **	0.09	0.85	0.01	0.00
**I1NFV9**	Uncharacterized protein	n.a.	lipid metabolic process (GO:0006629)	** **	0.39	0.70	0.22	0.01
**I1JCB6**	Uncharacterized protein/Endochitinase	External stimuli response	n.a.	** **	0.12	0.79	0.20	0.05
**I1KCB4**	Uncharacterized protein	n.a.	n.a.	** **	0.31	0.83	0.14	0.00
**C6SWY6**	Uncharacterized protein/Protein LlR18B	External stimuli response	defense response (GO:0006952)	**x**	0.21	0.76	0.17	0.00
**Q9FQD4**	Glutathione S-transferase	Protein modification	glutathione metabolic process (GO:0006749)	**x**	0.64	1.00	0.07	0.01
**I1MGK2**	Bet v I/Major latex protein	External stimuli response	defense response (GO:0006952)	**x**	0.80	1.00	0.03	0.01
**I1L0V9**	Bet v I/Major latex protein domain-containing protein	External stimuli response	defense response (GO:0006952)	**x**	0.39	1.00	0.05	0.00
**A0A0R0JSG7**	Peroxidase	Redox homeostasis	response to oxidative stress (GO:0006979)	**x**	0.80	1.00	0.13	0.02
**A0A0R0J536**	1-aminocyclopropane-1-carboxylate oxidase homolog 4	Enzyme classification	n.a.	** **	0.40	0.85	0.12	0.00
**A0A0R0G7L1**	Xylogen-type arabinogalactan protein	Cell wall	n.a.	** **	0.39	0.77	0.10	0.00
**C6TFP9**	Non-specific lipid-transfer protein	Lipid metabolism	lipid transport (GO:0006869)	** **	1.00	1.00	0.13	0.08
**A0A0R4J3X1**	Uncharacterized protein	Lipid metabolism	n.a.	** **	0.63	0.98	0.19	0.00
**I1JUR2**	BURP domain protein RD22	External stimuli response	n.a.	** **	0.25	0.14	0.07	0.00
**A1KR24**	Dehydrin	External stimuli response	n.a.	** **	0.07	0.41	0.02	0.00
**I1JWI5**	Uncharacterized protein	External stimuli response	n.a.	** **	0.18	0.11	0.27	0.00
**K7M9X3**	Uncharacterized protein	External stimuli response	n.a.	** **	0.37	1.00	1.93	0.06
**I1ME53**	Uncharacterized protein	External stimuli response	response to biotic stimulus (GO:0009607)	** **	0.56	0.99	1.70	0.10
**A0MZ84**	non-haem ferritin	Nutrient uptake	n.a.	** **	3.63	1.00	5.39	0.00
**R9ZS06**	Ribulose bisphosphate carboxylase large chain	Photosynthesis	n.a.	** **	1.02	1.00	2.86	0.38
**A1YZE0**	Granule-bound starch (amylose) synthase	Carbohydrate metabolism	starch biosynthetic process (GO:0019252)	** **	1.23	0.85	5.14	0.26
**C6SY10**	GASA precursor polypeptide	Phytohormones	n.a.	** **	7.42	0.01	8.71	0.82
**C6T9R8**	glyceraldehyde 3-phosphate dehydrogenase	Photosynthesis	glucose metabolic process (GO:0006006)	** **	2.89	0.04	3.91	0.70
**(B) Root Proteins PC1 cutoff <0.65**								
**I1LEI5**	Uncharacterized protein	External stimuli response	n.a.	** **	0.09	0.02	0.03	0.00
**I1KHQ7**	FAD-binding PCMH-type domain-containing protein	oxidases - copper, flavone etc.	L-ascorbic acid biosynthetic process (GO:0019853)	** **	0.08	0.00	0.06	0.00
**I1LVB1**	Alpha-amylase/subtilisin inhibitor	protease/enzyme inhibitor	n.a.	** **	0.06	0.00	0.03	0.00
**I1NAC8**	Alpha carbonic anhydrase 7	Enzyme classification	n.a.	** **	0.05	0.02	0.02	0.00
**I1K6M2**	Protein P21	External stimuli response	defense response (GO:0006952)	**x**	0.09	0.16	0.02	0.02
**I1L1I7**	tryptophan aminotransferase	amino acid metabolism	amino acid metabolic process (GO:0006520)	** **	0.01	0.00	0.01	0.00
**B1ACD2**	Putative protease inhibitor	protease/enzyme inhibitor	response to wounding (GO:0009611)	**x**	0.02	0.00	0.01	0.00
**I1K2W8**	Berberine bridge enzyme-like 28	Enzyme classification	n.a.	** **	0.05	0.00	0.03	0.00
**I1J7M4**	Peroxidase	Redox homeostasis	response to oxidative stress (GO:0006979)	**x**	0.04	0.00	0.03	0.00
**C6T3N0**	Inhibitor of trypsin and hageman factor	External stimuli response	response to wounding (GO:0009611)	**x**	0.02	0.01	0.01	0.00
**C6T376**	Class-10 pathogenesis-related protein 1	External stimuli response	n.a.	** **	0.03	0.01	0.02	0.01
**I1MZT4**	Peroxidase	Redox homeostasis	response to oxidative stress (GO:0006979)	**x**	0.07	0.00	0.05	0.00
**I1L927**	Uncharacterized protein	External stimuli response	n.a.	** **	0.12	0.00	0.04	0.00
**I1M395**	Knot1 domain-containing protein	External stimuli response	n.a.	** **	0.06	0.45	0.02	0.01
**I1L2V6**	Pectinesterase inhibitor 7	protease/enzyme inhibitor	negative regulation of catalytic activity (GO:0043086)	** **	0.02	0.46	0.01	0.01
**I1MPC5**	Acidic endochitinase	External stimuli response	n.a.	** **	0.03	0.04	0.03	0.00
**I1JKZ9**	Peroxidase	Redox homeostasis	response to oxidative stress (GO:0006979)	**x**	0.01	0.01	0.02	0.00
**I1JCB6**	Endochitinase	External stimuli response	n.a.	** **	0.07	0.57	0.01	0.04
**K7M2U5**	Uncharacterized protein	signaling	n.a.	** **	0.11	0.01	0.03	0.00
**I1LAF3**	SBT4 protease	Protein degradation	proteolysis (GO:0006508)	** **	0.03	0.05	0.01	0.00
**I1MG80**	Berberine bridge enzyme-like 8	Enzyme classification	n.a.	** **	0.09	0.01	0.05	0.00
**I1LC49**	pepsin-type protease	Protein degradation	proteolysis (GO:0006508)	** **	0.03	0.32	0.01	0.00
**I1JBQ0**	Hypersensitive-induced response protein	External stimuli response	n.a.	** **	0.22	0.56	0.02	0.00
**C7S8D4**	Germin-like protein 20	External stimuli response	n.a.	** **	0.15	0.81	0.02	0.00
**I1M587**	Endochitinase PR4	External stimuli response	polysaccharide catabolic process (GO:0000272)	** **	0.04	0.00	0.05	0.01
**I1KW53**	Uncharacterized protein	protease/enzyme inhibitor	n.a.	** **	0.04	0.00	0.07	0.01
**I1MI59**	Uncharacterized protein	protease/enzyme inhibitor	n.a.	** **	0.03	0.00	0.10	0.00
**I1MV71**	beta-like-type expansin	Cell wall	anatomical structure morphogenesis (GO:0009653)	** **	0.08	0.00	0.05	0.00
**I1LVB0**	Uncharacterized protein	protease/enzyme inhibitor	n.a.	** **	0.07	0.00	0.05	0.00
**I1ME54**	Uncharacterized protein	External stimuli response	response to biotic stimulus (GO:0009607)	** **	0.08	0.03	0.04	0.05
**C6TCC9**	Uncharacterized protein	protease/enzyme inhibitor	response to wounding (GO:0009611)	**x**	0.02	0.00	0.03	0.00
**I1KW54**	Alpha-amylase/subtilisin inhibitor	protease/enzyme inhibitor	n.a.	** **	0.04	0.00	0.04	0.00
**C6TI83**	Uncharacterized protein	External stimuli response	n.a.	** **	6.23	0.00	55.75	0.00
**C6T551**	Uncharacterized protein	External stimuli response	defense response (GO:0006952)	** **	9.69	0.00	19.84	0.00
**A0A0R0EY71**	Secoisolariciresinol dehydrogenase	oxidoreductase activity	n.a.	** **	13.79	0.00	22.21	0.00
**I1LCN2**	virus infection resistance factor (BTR1)	External stimuli response	n.a.	** **	12.46	0.00	12.97	0.00

Soybean plants (Glycine max cv Primus) were grown semi-hydroponically without (Ctrl.) or with 0.5 mM tungsten (W) in the nutrient solution and with two different nitrogen supply regimes (N fix: inoculated with B. japonicum, week 1&2 0.25 mM KNO3 & week 3-5 zero N; N fed: week 1–5 10 mM KNO3). Values shown are ratios of control treatments/tungsten treatments (n = 3-4). Proteins were considered significantly changed when the change (ratio) between compared treatments was higher or at least 2-fold as well as a p-value below 0.05. GO terms (Biological function) significantly enriched (sig. E.) are indicated with an x.

#### 3.3.4 Root proteome

Of 3,518 identified proteins in roots, 2,058 were detected in at least three out of four replicates. Of those 1,015 showed a significantly changed abundance due to tungsten exposure, of which half (47.6%) were exclusively affected in N fix plants (**Supplementary Figure S4**). Another, 26.5% were exclusively changed in N fed roots and 25.9% were changed in N fed as well as in N fix plants (**Supplementary Figure S4**). Of the 746 proteins that significantly changed in the tungsten-exposed N fix plants compared to the respective control, 458 showed a decrease and 289 an increase of abundance. In the N fed treatment, more than two-thirds of the significantly changed proteins were depleted (385/147). A full list of significantly changed root proteins can be found in the supplements (**Supplementary Table S6**).

The difference between tungsten-stressed plants and controls (PC1) was determined by proteins involved in “external stimuli response” [endochitinases, protein P21, knot1 domain-containing protein, hypersensitive-induced response protein (HIR) and pathogenesis-related proteins] and “protease/enzyme inhibitor” (inhibitor of trypsin and hageman factor, alpha-amylase/subtilisin inhibitor, and pectinesterase inhibitor 7) (**Figure 5**; **Table 3B**). Additionally, “protein degradation” (SBT4 protease and pepsin-type protease), “redox homeostasis” (peroxidases), proteins with “enzyme classification” (Berberine bridge enzyme-like 8 and 28 and alpha carbonic anhydrase 7) as well as “amino acid metabolism” (tryptophan aminotransferase) were functional categories that showed the highest loadings (**Table 3B**). The enrichment analysis of proteins with highest loadings for PC1 ( ± 0.05) revealed the biological processes “response to stimulus (GO:0050896)”, “response to oxidative stress (GO:0006979)”, “reactive oxygen species metabolic process (GO:0072593)”, “cellular response to chemical stimulus (GO:0070887)”, and “defense response (GO:0006952)” as well as their subordinate terms to be significantly enriched (**Table 3B**; **Supplementary Table S7C**).

## 4 Discussion

### 4.1 Enhanced tungsten stress resilience through symbiont-induced production of protective compounds

Heavy metal ions are known to cause oxidative damage by disrupting cellular processes and enzymatic activity, binding of glutathione and -SH groups of proteins, and displacing nutrients from binding sites as well as partaking in Fenton or Fenton-like reactions (Sharma and Dietz, 2009; Hossain and Komatsu, 2012). This has detrimental effects on cellular redox homeostasis, leads to lipid peroxidation, and disrupts membrane integrity as well as damages proteins and DNA (Michalak, 2006). We have previously found evidence that, similarly to other heavy metals, tungsten interferes with oxidative homeostasis and leads to an increased production of reactive oxygen species (Preiner et al., 2019). Plants combat oxidative stress by keeping the mitochondrial electron transport chain sufficiently oxidized, enhancing the biosynthesis of antioxidative enzymes such as catalase (CAT), superoxide dismutase (SOD), ascorbate peroxidase (APX), peroxidase (PX), and lipoxygenase (LOX), as well as by increasing the production of non-enzymatic antioxidative metabolites such as phenols, flavonoids, and organic acids (Møller, 2001; Gratão et al., 2005).

In this study, we found further evidence that tungsten exposure leads to oxidative stress and that tungsten stress response involves a significant readjustment of the antioxidative system. The GO enrichment analysis revealed that proteins involved in response to oxidative stress (GO:0006979) as well as oxygen species metabolic processes (GO:0072593) were among the proteins contributing most to the difference between the controls and tungsten-treated plants as well as between the different N regimes (**Supplementary Table S7**). Additionally, the leaves of N fix plants showed significantly elevated levels of peroxidases, glutathione peroxidase, glutathione reductase, superoxide dismutase (Cu–Zn), and H-type thioredoxin (**Supplementary Tables S5**, **S6**) as well as a markedly lower IC50 value (**Figure 2**). This suggests an enhanced antioxidative capacity of symbiotically grown plants during tungsten exposure. Consistent with this, our data clearly shows that symbiotically grown soy plants show a greater capacity to actively synthesize certain primary and secondary metabolites such as gluconic acid, polyamines, phenolic compounds (flavonoids and tannins elevated but not significant), and proline as well other amino acids to mitigate the deleterious effects of tungsten on plant redox homeostasis (**Figures 2**, **4**). Additionally, our findings indicate that these protective compounds play a role in the chelation and reduction of root-to-shoot trafficking of tungsten, as translocation to leaves was significantly higher in N fed plants compared to that in N fix plants (**Table 2**).

In leaves, phenolic compounds seem to play a key role in the observed symbiont-specific tungsten stress response (**Figure 2**). On the one hand, phenolic compounds serve as important non-enzymatic radical scavengers by directly trapping reactive oxygen and nitrogen species and acting as electron donors to peroxidases, mediating the formation of less reactive phenoxyl radicals (Sakihama et al., 2000; Winkel-Shirley, 2002; Maksymiec, 2007; Sytar et al., 2013). On the other hand, flavonoids have been shown to be involved in the chelation and vacuolar sequestration of heavy metals, potentially reducing the toxic effects on cellular metabolism and redox homeostasis. Hale et al. (2001, 2002) showed that anthocyanins are able to chelate not only molybdenum but also tungsten by forming complexes and thus facilitate tungsten accumulation in brassica. Additionally, Xu et al. (2018) found that increased concentrations of flavonoids (quercetin, glycitein, trans-resveratrol, p-coumaric acid, and glycinol) contribute to tolerance to excess molybdenum (100 mg L^-1^) in 3-week-old soybean seedlings.

Apart from phenolic compounds, the principal component analysis revealed a possible role of polyamines in tungsten stress response, as they were among the metabolites with the highest loadings (PC1) separating tungsten-treated N fix plants from all the other treatments (**Figure 4**). Polyamines are low-molecular-weight polycationic amines that are thought to be involved in an array of diverse metabolic processes such as stabilizing membranes and nucleic acids, modulating enzyme activity and scavenging free radicals as well as regulating development, plant growth, protein biosynthesis, and cell division (Walters, 2000; Menéndez et al., 2019; Hidalgo-Castellanos et al., 2022). Although the contribution of polyamines to abiotic stress response in plants is only partially understood, their levels and the activities of enzymes involved in their biosynthesis have been observed to be enhanced in response to various types of stress (Evans and Malmberg, 1989). As comprehensively reviewed by Menéndez et al. (2019), in the context of heavy metal stress, polyamines might play a role in protecting membrane systems and cellular processes from oxidative damage induced by redox-active metal ions. Interestingly, the exogenous application of spermidine to mung bean led to reduced uptake and translocation of Cd as well an increase of antioxidant enzyme activity and levels of non-enzymatic radical scavengers, which effectively prevented oxidative damage and chlorophyll degradation (Nahar et al., 2016; Menéndez et al., 2019). Similarly, the observed increase of putrescine and spermidine concentrations in the leaves of tungsten-stressed N fix plants (**Figure 4**) might help to reduce oxidative stress and stabilize membrane systems and cellular processes.

While changes in phenol and polyamine levels were primarily found in leaf tissue (**Figure 4**), we observed significant alterations in the abundance of certain enzymes involved in the glutathione–ascorbate cycle (glutathione reductase, glutathione peroxidase, and glutathione S-transferase) and of certain antioxidative enzymes (peroxidases and catalases) in the roots of N fix plants (**Supplementary Table S6**). This could be the result of an adaptive response to bacterial symbiosis, as reactive oxygen species and the plant’s antioxidative defense system play a crucial role in nodule formation as well as the establishment and functioning of symbiosis (Stambulska et al., 2018). As nodules are thought to mitigate stress by serving as an important sink for heavy metals, an effective antioxidative system is necessary to protect nitrogenase from oxidative damage (Stambulska et al., 2018). In addition to this enzyme-mediated antioxidative response, gluconic acid was significantly increased in the roots of tungsten-stressed plants of both N regimes (**Figure 4**). However, this accumulation was again more prominent in the roots of symbiotically grown plants (50-fold increase) compared to the N fed treatment (**Figure 4**; **Supplementary Table S4**). The inoculation with gluconic acid-producing bacteria has been shown to enhance plant growth and restore root elongation of cadmium (Cd^2+^)-stressed plants (Kavita et al., 2008). Furthermore, it improves the bioavailability and uptake of phosphate and certain trace elements while alleviating the symptoms of heavy metal toxicity by forming stable complexes (Stella and Halimi, 2015; Mandal et al., 2016; Wood et al., 2016). Research by Xu et al. (2018) found more than 107-fold increase in gluconic acid concentrations in the roots of soybean plants (non-symbiotic) exposed to excess molybdenum. They hypothesize that, together with other organic acids, gluconic acid reduces molybdenum toxicity by chelating molybdenum and thus hindering root-to-shoot trafficking of the transition element. Interestingly, gluconic acid has been found to form stable complexes not only with molybdenum (VI) but also tungsten (VI) in aqueous solutions (Llopis et al., 1986; Ramos et al., 1997).

**Figure 6:**
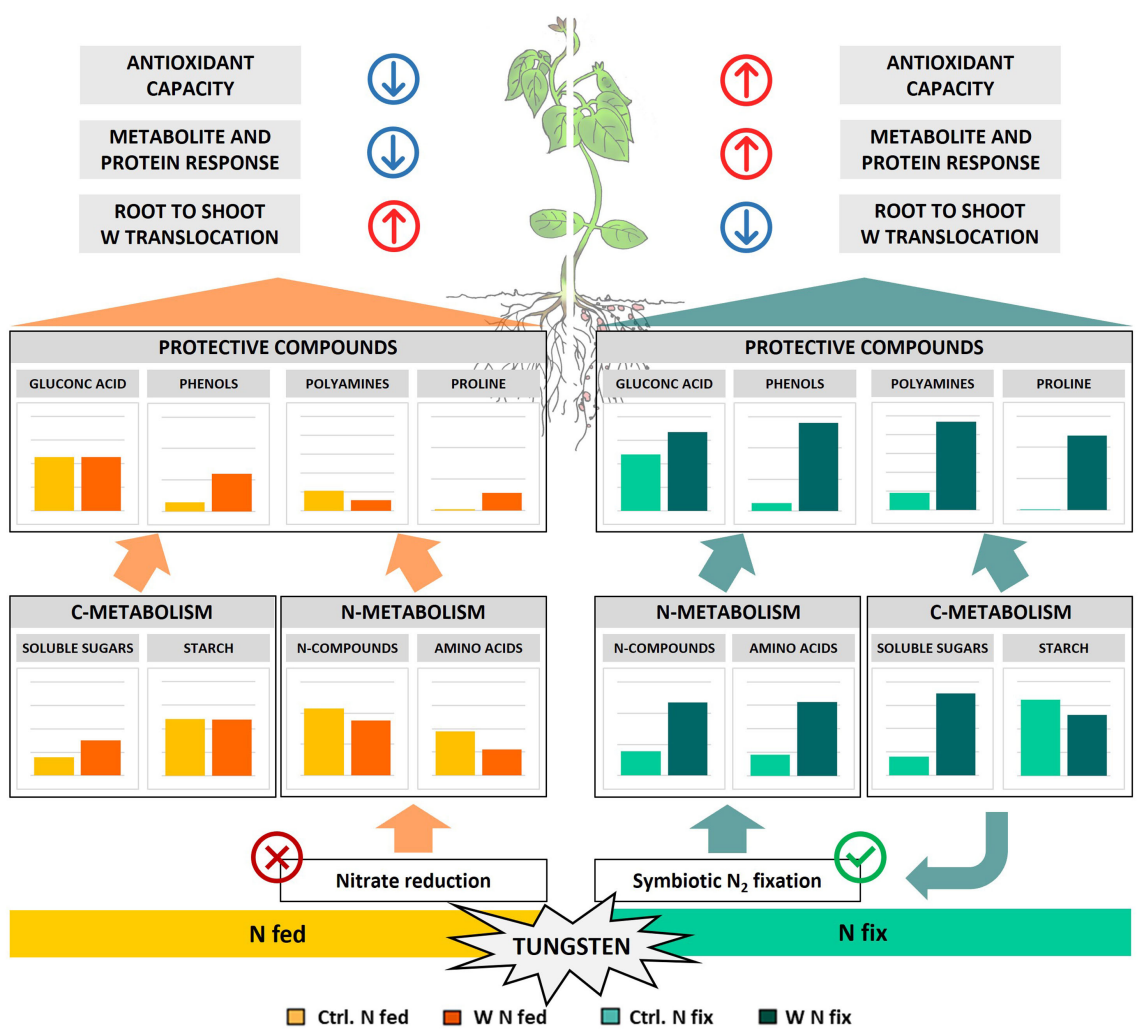
Schematic representation of metabolic tungsten stress response of soybean plants (*Glycine max* cv Primus) grown semi-hydroponically without (Ctrl) or with 0.5 mM tungsten (W) and two different nitrogen supply regimes (N fix: inoculated with *B. japonicum*, weeks 1 and 2: 0.25 mM KNO_3_ and weeks 3–5: zero N; N fed: weeks 1–5: 10 mM KNO_3_).

The idea that tungsten uptake and translocation within the plant is affected by protective compounds such as polyamines and gluconic acid is supported by our results. Tungsten-stressed N fix plants, which showed the highest fold changes of these metabolites, exhibited a significantly lower root-to-shoot translocation of tungsten (**Table 2**). Additionally, our proteomic data revealed significant alterations of cell wall metabolism in the roots to facilitate tungsten sequestration and mitigate the deleterious effects of heavy metal stress. Two proteins with some of the highest loadings of PC1, Berberine bridge enzyme-like proteins 8/28 (I1MG80 and I1K2W8), are interesting in this respect (**Figure 5**, **Table 3B**). These enzymes belong to the FAD-linked oxidase superfamily and are involved in the inactivation of oligogalacturonides, which are cell wall fragments that serve as elicitors of defense response and seem to regulate plant growth (auxin antagonist) and cell wall formation/degradation (Ferrari et al., 2013). Oligogalacturonide (OG) accumulation as well as external application has been found to result in reduced growth, disturbed development, necrosis of tissue, and, eventually, plant death (Chen et al., 2022). Berberine bridge enzyme-like proteins have been found to oxidize OGs and thus protect cell walls from degradation and maintain plant growth, as the H_2_O_2_ formed during OG oxidation can be used by peroxidases to reinforce cell walls (Chen et al., 2022; Scortica et al., 2023). Interestingly, peroxidases were also among the proteins with the highest loadings in PC1 (I1J7M4 and I1JKZ9) (**Figure 5**; **Table 3B**). Our results indicate that the immobilization of tungsten in the root system, together with the observed changes in the antioxidative system as well as the enhanced production of protective compounds (polyamines, gluconic acid, and phenols), potentially resulted in reduced oxidative damage in N fix plants (**Figure 6**).

### 4.2 Maintenance of N metabolism in tungsten-exposed N fix plants

Heavy metal stress interferes with plant metabolic pathways on various levels. One hallmark of heavy metal exposure is the synthesis and accumulation of various N-containing metabolites such as small peptides, amino acids, and polyamines with the potential to mitigate the heavy metal toxicity (Sharma and Dietz, 2006). Among various N-containing protective metabolites that showed significantly increased concentrations in the tungsten-stressed N fix plants, proline had the highest loadings (PC1) and highest fold change (100-fold in leaves and 500-fold in roots) (**Figure 4**). The accumulation of proline is well documented in response to various types of biotic and abiotic stresses including pathogen attack, salinity, drought, flooding, and heavy metal stress (Spormann et al., 2023). Similarly, a variety of roles such as antioxidative, osmoregulatory, or signaling function have been attributed to proline during stress (Spormann et al., 2023). High constitutive proline contents found in taxonomically distant metal-tolerant plant species suggests a function of proline in metal stress tolerance (Schat et al., 1997; Sharma and Dietz, 2006; Szepesi and Szőllősi, 2018). It is thought to play a role in stabilizing cellular homeostasis, providing reducing equivalents for mitochondrial respiration and protecting enzymatic function during stress as well as binding and chelating heavy metals (Hare and Cress, 1997; Sharma and Dietz, 2006; Szabados and Savouré, 2010; Szepesi and Szőllősi, 2018). Proline accumulation during stress is thought to be driven by proteolysis and increased synthesis as well as reduced catabolism (Szabados and Savouré, 2010; Spormann et al., 2023). We not only found a pronounced increase in proline levels but we also observed significantly higher levels of P5CS, a central enzyme of proline biosynthesis, in N fix roots (**Supplementary Table S6**). Together with higher levels of proline precursors such as glutamate and ornithine (**Supplementary Table S4B**), this indicates an enhanced proline biosynthesis in symbiotically grown soy plants. Although the proline levels were also increased in N fed plants, the magnitude of the observed increase of proline concentrations in N fix plants (more than 100-fold) points to a specific symbiosis-induced response (**Figure 4**; **Supplementary Tables S3B**, **S4B**). Interestingly, Selim et al. (2021) found that inoculation with actinobacteria ameliorates the adverse effects of tungsten nanoparticles on legumes (alfalfa, pea, and soybean) by the accumulation of proline, soluble sugars, and polyamines.

To ensure a sufficient N supply for the biosynthesis of proteins and protective N compounds such as proline and polyamines, N assimilation and subsequent metabolic pathways play a critical role in heavy metal tolerance and stress response (Sharma and Dietz, 2006). In the context of tungsten stress, this seems especially interesting as it has been shown that a major component of tungsten toxicity in plants is the inhibition of nitrogen metabolic processes by substitution of the molybdenum ion bound to the enzymatic co-factor of nitrate reductase (Harper and Nicholas, 1978; Deng et al., 1989). The picture seems not to be as clear for the Fe–Mo cofactor containing bacterial nitrogenase utilized by symbiotic rhizobia. Some studies have shown that bacterial nitrogenase remains functional upon tungsten exposure (Kletzin and Adams, 1996; Schwarz et al., 2007; Oburger et al., 2018; Preiner et al., 2019).

In this experiment, we therefore exposed plants to tungsten only after the establishment of rhizobium symbiosis, when nodules were fully developed. Although tungsten clearly affected plant growth, photosynthesis, and biomass (**Table 1**), we found no effect on nodule number (**Table 1**). Together with our previous findings (Oburger et al., 2018; Preiner et al., 2019) as well as the increased concentrations of nitrogen compounds such as ureides (allantoin and allantoic acid) and amino acids, this can be seen as further evidence for maintained nitrogen fixation activity during tungsten exposure (**Figure 4**; **Supplementary Tables S3**, **S4**). As discussed above, we also found a systemic metabolic readjustment clearly connected to the symbiosis with N_2_-fixing bacteria, which enabled the plants to synthesize a variety of protective metabolites such as amino acids and polyamines as well as phenolic compounds (**Figures 2**, **4**).

The sustained or even increased concentrations of allantoin and allantoic acid as well as the amino acids glutamine (gln) and glutamic acid (glu) in the roots and leaves of tungsten-stressed N fix plants (**Figure 4**) suggest that sufficient nitrogen (N) for the production of amino acids and protective compounds is provided by either N_2_ fixation or a significant reduction of ureide catabolism (Ohyama et al., 2017). In ureidic legumes such as soybeans, ammonia from N_2_ fixation is rapidly assimilated into glutamine and subsequently to glutamate by glutamate synthetase (GOGAT); the fixed nitrogen is then used for *de novo* synthesis of purine nucleotides, which then is metabolized via uric acid to allantoin and allantoic acid, which represent the main forms of transport of fixed nitrogen from root nodules to leaf tissue (Ohyama et al., 2017; Ono et al., 2021). Indeed we found key enzymes involved in ureide biosynthesis (glutamate dehydrogenase, glutamate synthetase, glutamine synthetase, uricase, hydroxyisourate hydrolase, and allantoinase) as well as ureide catabolism (urease and ureidoglycolate amidohydrolase) to be significantly more highly abundant in the roots of tungsten-treated N fix plants compared to the control (**Supplementary Table S6**). Additionally, no significant reduction of ureide catabolic enzymes was observed in the leaves of N fix plants, where the highest levels of free amino acids and ureides were measured. Together with the reduction of several TCA cycle organic acids in leaves (**Supplementary Tables S3**, **S4**), this suggests that the ureides were transported from the root nodules to the sink organs where they were used for amino acid biosynthesis. The notion that the observed metabolic changes are a result of maintained N_2_ fixation rather than ureide catabolism is also supported by our previous results, which showed that tungsten does not directly affect N_2_ fixation rate (activity per nodule) but rather hampers nodule formation and development (Oburger et al., 2018; Preiner et al., 2019).

The increase in asparagine (asn), glutamic acid (glu), and glutamine (gln) concentrations (**Supplementary Tables S3**, **S4**) in N fix plants also indicates enhanced protein turnover and recycling of amino acids as well as hints at the function of these amino acids as transient N storage (Carter and Tegeder, 2016; Ohyama et al., 2017). This is supported by the fact that despite significantly higher levels of proteolytic enzymes as well as protease inhibitors in leaves (**Table 3**), the overall protein concentrations remained comparable in tungsten-stressed N fix plants (**Table 1**). Together with the increase in abundance of numerous proteins, especially of proteins with stress-responsive, antioxidative, and enzymatic function in N fix plants upon stress (**Supplementary Tables S5**, **S6**), this indicates that the significant amino acid accumulation is a result of an overall metabolic readjustment of N fix plants upon tungsten stress.

In N fed plants, conversely, the lower levels of nitrogen compounds (allantoin, allantoic acid, and asparagine) (**Supplementary Tables S3**, **S4**) as well as the depletion of enzymes involved in nitrate assimilation (nitrite reductase) (**Supplementary Table S6**) in the tungsten treatment show the direct inhibitory effect of tungsten on nitrate reduction. This is furthermore supported by previous research where the direct inhibitory action of tungsten on nitrate reductase activity was shown (Nicholas et al., 1976; Deng et al., 1989; Oburger et al., 2018; Preiner et al., 2019).

Taken together, our data supports the idea that the increase in amino acids and protective N-metabolites is fueled by ureide *de novo* biosynthesis and consequently N_2_ fixation by associated rhizobia rather than by a reduction of ureide catabolism or enhanced proteolytic activity. Nitrogen-fertilized plants (N fed) conversely did not possess the capacity to respond to tungsten-induced oxidative stress in the same way that the assimilation of nitrogen via nitrate reduction was inhibited by tungsten (**Figure 6**).

### 4.3 Symbiotically induced carbohydrate allocation provides carbon backbones for the production of protective compounds

Carbohydrate metabolism is highly sensitive to environmental stress. Heavy metals are known to affect carbohydrate metabolism by disrupting photosynthesis, source-to-sink transport, and enzyme activities as well as respiration and thus have a detrimental effect on plant growth, seed development, and productivity (Samarakoon and Rauser, 1979; Viehweger, 2014).

Although no changes in photosynthetic pigment contents were observed (**Supplementary Table S1A**), our results clearly show that tungsten impairs the photosynthetic activity and efficiency of PSII (**Supplementary Figure S1**; **Supplementary Table S1B**). Additionally, it led to a reduction of proteins of the photosynthetic apparatus in both N regimes (**Supplementary Table S5**). Interestingly, symbiotically grown tungsten-stressed plants exhibited a distinct metabolic response compared to their N fed counterparts (**Figure 6**). Despite a reduced photosynthetic activity, we found significantly higher levels of soluble carbohydrates (glucose, fructose, galactose, and raffinose) and a notable reduction of starch contents in the roots and leaves of tungsten-stressed N fix plants (**Figure 4**; **Supplementary Tables S3A**, **S4A**). In N fed plants, this increase was less pronounced, and the starch concentrations were not affected or even slightly elevated (**Supplementary Tables S3**, **S4**). Our proteomic analysis revealed that in the leaves of tungsten-stressed N fix plants, granule-bound starch synthase (A1YZE0) was markedly reduced and among the proteins with the highest negative loadings in PC1 (**Figure 4**). Furthermore, alpha- and beta-amylases and the levels of sucrose synthase were significantly increased in the leaves and roots of tungsten-stressed N fix plants (**Supplementary Table S5**). Interestingly, a reduction of sucrose synthase activity has been shown to be responsible for the inhibition of nitrogen fixation during drought in *Medicago truncatula* (Larrainzar et al., 2007). The instance that we found a significantly increased abundance of sucrose synthase in tungsten-stressed N fix plants indicates that the carbohydrate allocation found might also serve to “feed” bacterial symbionts and again points to a maintenance of N_2_ fixation. The reduced starch content in tungsten-stressed N fix plants partially contradicts our previous results (Preiner et al., 2019), where we observed increased starch concentrations in whole shoot tissue of both N regimes. However, it is important to note that, in this study, only leaf tissue was used for starch content measurement. Overall, our findings suggest that the suppression of starch synthesis, together with enhanced sucrose synthesis, resulted in the accumulation of soluble carbohydrates in N fix plants. Starch depletion, paired with an increase of soluble carbohydrates during the day, when usually transitory starch concentrations increase due to photosynthetic activity, has been observed in plants growing under various adverse conditions (Thalmann and Santelia, 2017). When photosynthetic activity is reduced by environmental constraints, starch is degraded in order to ensure a supply of soluble carbohydrate to maintain cellular respiration and water potential and to protect metabolic processes from oxidative stress (Thalmann et al., 2016). Additionally, certain sugars such as sucrose might also indirectly enhance the antioxidative capacity of plants by acting as secondary messengers that influence the photosynthetic activity as well as the expression of radical scavengers such as anthocyanins (Van den Ende and El-Esawe, 2014).

Apart from their central role as energy source and osmolyte as well as structural component of plant cells, soluble carbohydrates also serve as the carbon backbone of secondary metabolites and protective compounds such as phenols, polyamines, and amino acids (Jeandet et al., 2022). The evident symbiont-specific investment in the synthesis of amino acids and protective compounds thus depends not only on a constant supply of N but also on the significant incorporation of carbon. Our results clearly show that N fix plants underwent a significant metabolic readjustment to provide carbon sources for nitrogen fixation as well as for the biosynthesis of secondary metabolites and protective compounds (**Figure 6**). We believe that the increased investment in stress alleviation, together with reduced photosynthesis rates, most likely resulted in the mobilization of carbon storage pools in N fix plants in an effort to maintain cellular respiration and osmotic balance. A similar interplay of transitory starch, soluble carbohydrates, and protective compounds such as proline has been reported in drought-stressed *Arabidopsis* (Zanella et al., 2016).

## 5 Conclusion

Taken together, this study presents strong evidence that rhizobia symbiosis has an ameliorating effect on tungsten toxicity. Although tungsten interferes with the integral parts of plant metabolism, hampering growth, photosynthesis, and development regardless of N supply, symbiotic plants were better equipped to maintain cellular respiration and oxidative balance. We believe that the maintenance of N_2_ fixation and allocation of carbohydrate storage pools enabled symbiotically grown plants to actively synthesize an array of metabolically expensive protective compounds such as polyamines, gluconic acid, and phenolic compounds and amino acids such as proline to mitigate the deleterious effects of tungsten on plants. This, in turn, resulted in the sequestration of tungsten in the root system as well as limited root-to-shoot trafficking. Additionally, the observed accumulation of soluble sugars in N fix plants provided reducing equivalents, energy, and osmotic adjustment in order to counterbalance the detrimental effects of tungsten on cell survival. Our findings underscore the importance of symbiotic relationships in enhancing plant resilience to tungsten-induced stress and highlight the complex mechanisms involved in plant adaptation to heavy metal stress.

## Data availability statement

The datasets presented in this study can be found in online repositories. The names of the repository/repositories and accession number(s) can be found below: ProteomeXchange Consortium, PXD047532.

## Author contributions

JP: Writing – review & editing, Writing – original draft, Visualization, Validation, Project administration, Methodology, Investigation, Formal analysis, Data curation, Conceptualization. IS: Writing – review & editing, Methodology, Formal analysis. EO: Writing – review & editing, Supervision, Resources, Project administration, Funding acquisition, Formal analysis, Conceptualization. SW: Writing – review & editing, Validation, Supervision, Resources, Project administration, Methodology, Investigation, Funding acquisition, Conceptualization.
