# Supplementary material for: Rhizobium symbiosis improves amino acid and secondary metabolite biosynthesis of tungsten-stressed soybean (Glycine max)
Source: Front Plant Sci. 2024 Apr 2;15:1355136. doi: 10.3389/fpls.2024.1355136 (PMC11020092; doi:10.3389/fpls.2024.1355136)
Supplement: Supplementary file 1 [file DataSheet_1.zip › SI/SI_1.pdf]

Supplementary Material

Rhizobium symbiosis improves amino acid and secondary metabolite biosynthesis of tungsten-stressed soybean (Glycine max)

Julian Preiner[1], Irene Steccari[1], Eva Oburger[2], Stefanie Wienkoop*[1],

[1] Molecular Systems Biology Unit, Department of Functional and Evolutionary Ecology, University of Vienna, Vienna, Austria

[2]Institute of Soil Research, Department of Forest and Soil Sciences, University of Natural Resources and Life Sciences Vienna, Tulln, Austria

*** Correspondence:**
Stefanie Wienkoop
stefanie.wienkoop@univie.ac.at

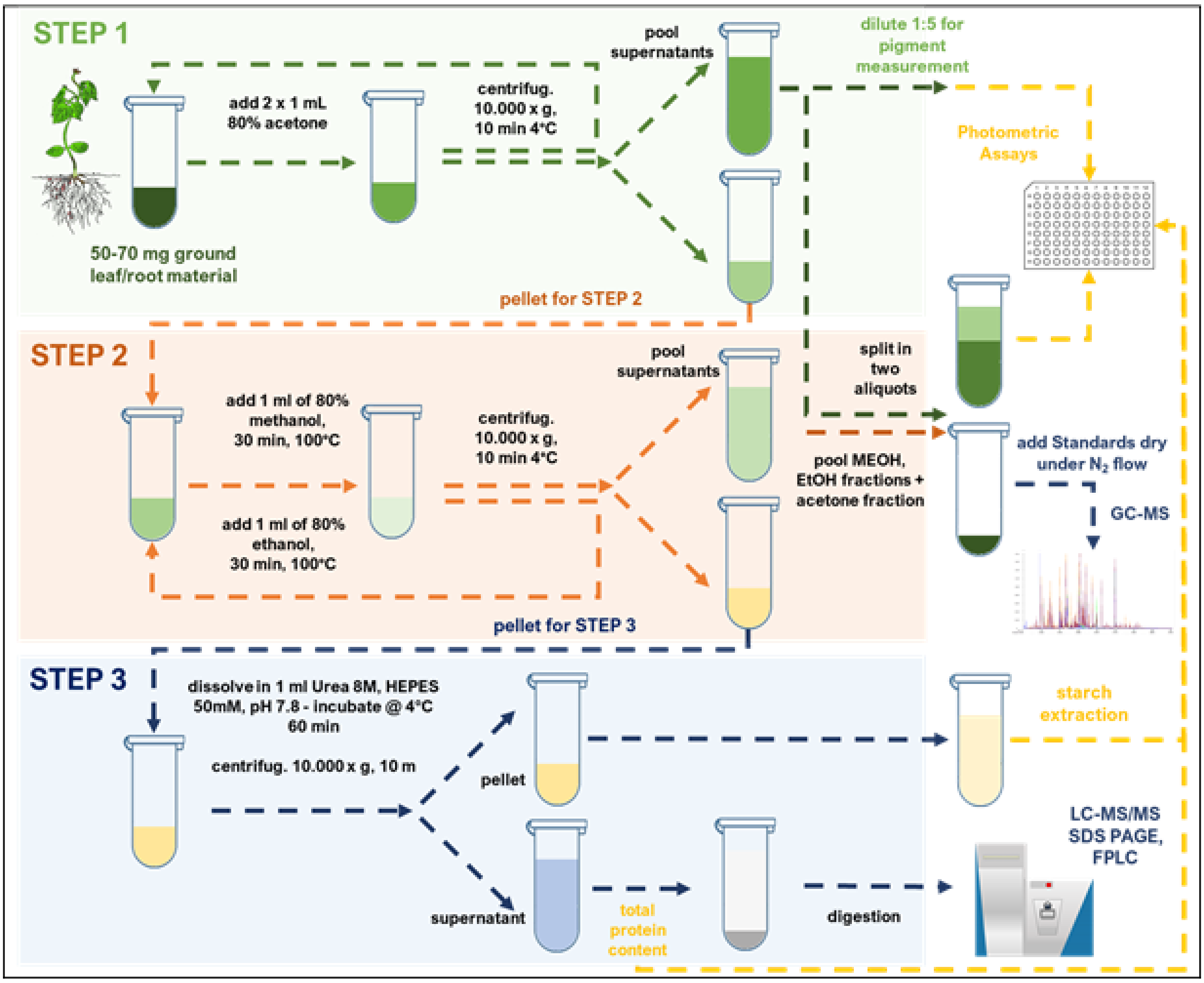

Sequential Extraction Protocol

Preparations: 3

STEP 1: Extraction of photosynthetic pigments according to (Lichtenthaler and Wellburn, 1983)..3

STEP 2: Extraction of soluble sugars, phenols and amino acids. 3

Sample preparation for GC-MS ANALYSIS 3

Sample preparation and protocols for Metabolic Marker Assay................................4

A. **Estimation of soluble carbohydrates Anthrone reagent** as per the method given by Hansen and Møller (1975)..5

B. **Estimation of free amino acids Ninhydrin method** given by Moore and Stein, (1948) Commercial Assay 6

C. **Estimation of phenols** according to John et al. (2014) and Rebaya et al. (2015) 7

D. **Estimation of flavonoids** Aluminium chloride method (Chang et al., 2002; Rebaya et al., 2015) 8

E. **Estimation of total condensed tannin** contents method (Broadhurst and Jones, 1978)..9

F. **Estimation of antioxidant activity (a):** Determination of DPPH radical-scavenging activity using the method described by Rebaya et al. (2015)...................10

STEP 3: Extraction of proteins and starch 11

A. **Protein Extraction** Adapted from Morgenthal et al. (2007); Nagler et al. (2015); Niessen et al. (2006)..11

B. **Extraction of starch from pellet (assay a)** (Nagler et al., 2015).....................12

STEP 4: Metabolite derivatization and measurement preparation: 14

Preparations:

Weigh in approximately 60 mg of finely ground plant material in eppis (keep on liquid nitrogen at all times!)
Note: To prevent samples from popping open during incubation/heating safe-lock eppis with safety clips or eppis with screwable lids should be used!
Note: all steps on ice (4°C)

STEP 1: Extraction of photosynthetic pigments according to (Lichtenthaler and Wellburn, 1983)

1. Add 1 ml of prechilled **80% acetone** to plant material, vortex thoroughly and put on ice.
2. Centrifuge at 10,000 x g for 10 minutes. (collect supernatants together in new 2 ml eppi)
3. Reextract the pellet in 0.5 ml of 80% acetone and centrifuge at 10,000 g for 10 minutes → **repeat step twice!** (**store pellet at 4°C for step 2**).
4. Collect all supernatants together in same eppi.
5. Take 200 µL of pooled samples and diluted to 1 mL with 80% acetone in 1.5 mL Eppendor tubes.
6. Use 250 µL of diluted samples (1:5) on to read absorbance at 663, 646, 470 nm with spectrophotometer (three replicates per sample) for estimation of pigments.

Calculations:
chlorophyll ***a (Ca)*** $(12.21A_{663}-2.81A_{646})*0.7$
chlorophyll **b *(Cb)*** $(20.13A_{646}-5.03A_{663})*0.7$
total amounts of carotenoids $((1000\ A_{470} - 3.27\ Ca - 104\ Cb)/229)*0.7$

When using a microplate and a sample volume of 250 µl, the extinction coefficient needs to be adjusted, based on the thickness of the layer in the respective type of microplate used. For the microplates used in our experiment (Greiner Bio-One, 96-Well microplate F-bottom), above formula was multiplied with 0.7 to adjust extinction coefficient for microplate. Concentrations are given in µg per mL. note: consider dilution factor for final concentration!

STEP 2: Extraction of soluble sugars, phenols and amino acids.

1. Re-extract pellet obtained in **STEP 1** in 1 mL **80% Methanol, vortex** and incubate at 95°C for 30 minutes.
2. CAUTION: Methanol is toxic! Use proper gloves, change immediately if contaminated! Work under ventilation hood.

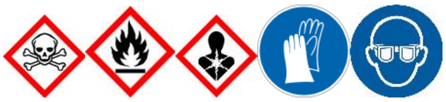

3. Cool content, centrifuge at 20,000 x g for 10 minutes
4. Re-extract pellet with 1 mL of **80% EtOH** and incubate at 95°C for 30 minutes
5. Cool content, centrifuge at 20,000 x g for 10 minutes
6. ***Remove solvents and dry pellets open under the hood at room temperature and store at -20°C until STEP 3***

Sample preparation for GC-MS ANALYSIS

For GC-MS analysis: Combine an aliqot of all supernatants as follows in a new 2 mL Eppendorf tube and dry down phases individually under the nitrogen flow.

Procede as follows:

- Take 1 mL of acetone phase **(STEP 1)**-> dry under nitrogen flow
- Add 5 µL internal standard for GC-MS (1 mM PGP/10 mM PE)
- Add 0.5 mL of methanol phase **(STEP 2)**-> dry under nitrogen flow
- Add 0.5 mL of ethanol phase **(STEP 2)**-> dry under nitrogen flow
- Store at -80°C until measurement.

All further steps for GC-MS analysis see ->STEP 4!

Sample preparation and protocols for Metabolic Marker Assay

For SEQ-Ex: Combine an aliqot of all supernatants as follows in a new 2 mL Eppendorf tube:

Procede as follows:

- Take 1 mL of acetone phase **(STEP 1)**
- Add 0.5 mL of methanol phase **(STEP 2)**
- Add 0.5 mL of ethanol phase **(STEP 2)**
- Use immediately to carry out photometric biomarker assays – if necessary can be stored at -80°C until measurement.

This mixture can be used for estimation of **total sugars, total phenols, total falvonoids and free amino acids.**

A. ***Estimation of soluble carbohydrates Anthrone reagent*** *as per the method given by Hansen and Møller (1975)*

Reagents Required:

- (D-glucose (5 mM Stock = 5 µmol/mL) in 80% ethanol). Prepare a standard curve in concentrations from 50 to 3000 nmol ml^{-1} and a blank throughout extraction and analysis procedure.)
- Prepare standard curve as follows (volumes in mL):

nmol ml^{-1}	50	250	500	1000	3000
Standard	0.01	0.05	0.1	0.2	0.6
ddH_2O	0.99	0.95	0.9	0.8	0.4

- pre-chilled 72% H_2SO_4 solution (100 mL -> 65.452 mL **98%** H_2SO_4)
  CAUTION: Sulfuric acid is strongly corrosive! Avoid contact with skin, clothes and pipettes. Use proper gloves, change immediately if contaminated and wash hands! Avoid inhaling. Use safety glasses. Use filter tips.

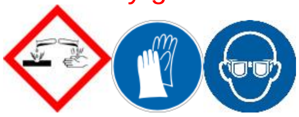

- anthrone reagent (2 g L^{-1} in conc. H_2SO_4, freshly prepared) ->): 0.5 g per 50 mL-> (for storage)-> then dilute 2.50 mL with 10 mL conc. H_2SO_4 fresh [or prepare fresh each time: 200 mg anthrone in 100 mL of ice-cold 95% sulphuric acid]

Procedure:

1. Dilute samples 50 µL of sample+50µL H_2O (2x dil.)
2. Mix 100 µL of diluted sample and 100 µL of standards (see above) and blank with 200 µL of pre-chilled 72% H_2SO_4 solution in a 1.5 mL Eppendorf tube and vortex.
3. Add 400 µL of the anthrone reagent
4. vortex
5. Heat at 95°C for 15 min.
6. After cooling on ice, vortex and read the absorbance in 2 replicates of 250 µL at 630 nm on spectrophotometer. Measure within 10 minutes after heating!
7. Background absorbance of the sample is read by reacting 100 µL sample with 600 µL 72% H_2SO_4 (without the anthrone reagent).

DISPOSAL:
Use pipette to empty 96-well plate. Collect waste in waste bottle:

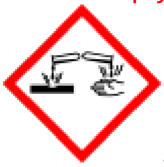

Sulfuric acid (H2SO4), aqueous solution maximal 72% H: 290-314 P:280-301+330+331-305+351+338-308+310

B. **Estimation of free amino acids Ninhydrin method** *given by Moore and Stein, (1948) Commercial Assay*

Reagents Required:

- Lysine stock solution (50 µM Lysine in 0,05% acetic acid [4.754 µL 99.8% acetic acid + 9.995 mL ddH_20]) were used to prepare standard curve. (0.0025 µmol to 0.05 µmol ml^{-1}). Add distilled water in all the test tubes to make up the volume to 0.2 mL (use 1.5 mL Eppendorf tubes).
- Prepare standard curve as follows (volumes in mL):

nmol ml^{-1}	10	20	30	40	50
Standard	0.04	0.08	0.12	0.16	0.2
ddH_2O	0.16	0.12	0.08	0.04	0
Ninhydrin reagent	0.1	0.1	0.1	0.1	0.1

- Ninhydrin commercial reagent (N7285 – sigma) -> always store under inert gas -> cover with N_2
- 95% ethanol.
- Distilled water.

Procedure:

1. Use 0.2 mL of sample/standard/blank and add 0.1 mL Ninhydrin reagent
2. Mix gently
3. Boil sample/standard at 95°C for 10 min
4. Rapidly cool down to room temp by placing them briefly on ice
5. Add 0.5 mL 95% ethanol to each tube
6. Mix contents by vortexing/shaking the tubes
7. Use 250 µL of each sample in 2 replicates and read absorbance of 570 nm
    a. If A570 exceeds 1.0, dilute samples with another volume of 95% ethanol.
    b. For std. solution: substract absorbance of standards and samples by absorbance of your blank (tube 1).

C. **Estimation of phenols** *according to John et al. (2014) and Rebaya et al. (2015)*

Reagents Required:

- Gallic acid (3,4,5-trihydroxybenzoic acid) 1 mg/mL stock. (100, 200, 300, 400, and 500 µg/ml) was used to prepare the standard curve.
- Prepare standard curve as follows (volumes in mL):

µg ml^{-1}	50	100	200	400	500
nmol	29.39	58.78	117.56	235.12	293.91
Standard	0.05	0.1	0.2	0.4	0.5
ddH_2O	0.95	0.9	0.8	0.6	0.5

- Folin-Ciocalteu phenol reagent
- 7% Na_2CO_3 solution
- Distilled water.

Procedure:

1. Add 100 µL of extracts or standard solutions to 700 µl of distilled water in 2 mL Eppendorf Tubes.
2. Prepare a reagent blank using distilled water
3. Add 80 µl of Folin-Ciocalteu phenol reagent to the mixture and shake.
4. After 5 minutes add 800 µl of 7% Na_2CO_3 solution to the mixture.
5. Incubate 90 minutes at room temperature
6. Method blank was prepared in the same manne but using ddH_2O instead of sample
7. Load 250 µL of each sample/standard/method blank in 2 replicates into microtiter wellplate and read absorbance at 550 nm.
8. Total phenolics content is expressed as nmol Gallic acid Equivalents (GAE)/mg FW

D. ***Estimation of flavonoids*** *Aluminium chloride method (Chang et al., 2002; Rebaya et al., 2015)*

Reagents Required:

- Quercetin (stock of 1g/L in 50% EtOH) 20, 40, 60, 80 and 100 µg/ml was used to prepare the standard curve ***(toxic!).***
  CAUTION: Quercetin is toxic if swallowed! Use nitrile gloves, change immediately if contaminated!

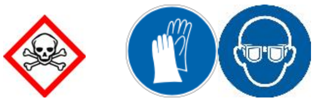

- Prepare standard curve as follows (volumes in µL):

µg ml^{-1}	20	30	60	80	100
nmol	6.61	9.92	19.85	26.46	33.08
Standard	20	30	60	80	100
ddH_2O	980	970	940	920	900

- 5% $NaNO_2$
- 10% $AlCl_3$
- 1M NaOH
- Distilled water.

Procedure:

1. Add an aliquot 100 µL of sample extracts or standard to 2 ml Eppendorf Tube containing 900 µl of distilled water or take 1 mL of the standards (prepared above)..
2. Add 60 µl of 5% $NaNO_2$
3. After 5 minutes, add 60 µl 10% $AlCl_3$
4. After 5 minutes, add 400 µl 1M NaOH and make up the volume to 2 ml with distilled water.
5. Method blank was prepared in the same manner but using ddH_2O instead of $NaNO_2$, NaOH and $AlCl_3$
6. Mix and load 250 µL of each sample/standard/blank in 2 replicates into microtiter wellplate and read absorbance against the blank at 510 nm.
7. The total flavonoid content is expressed as nmol quercetin equivalents (QE)/mg FW.

E. ***Estimation of total condensed tannin** contents method (Broadhurst and Jones, 1978)*

Reagents Required:

- Catechin (stock of 1g/L) 20, 40, 60, 80 and 100 µg/ml was used to prepare the standard curve
- Prepare standard curve as follows (volumes in µL):

$\mu g\ ml^{-1}$	20	40	60	80	100
nmol	6.88	13.78	20.67	27.56	34.44
Standard	20	40	60	80	100
ddH_2O	980	960	940	920	900

- Hydrochloric acid (conc.)
- Vanillin (4% in methanol)

Procedure:

1. An aliquot (200 µL) of extracts or (100 µL MQ+ 100 µL standard) was added to 1.5 mL Eppendorf Tube containing 750 µL of the vanillin solution and 350 µL of hydrochloric acid.
2. After 30 min of incubation at RT use 250 µL in 2 replicates in microtiter plate and read absorbance at 500 nm.

F. ***Estimation of antioxidant activity (a):*** *Determination of DPPH radical-scavenging activity using the method described by Rebaya et al. (2015)*

Reagents Required:

- ascorbic acid (3.0-15.0 µg.mL-1), gallic acid (0.5 to 5.0 µg.mL-1) were used to prepare the standard curve. Note: the standards are only needed if you want to compare your ROS activity (% inhibition) to that of the standards (see publication).
- DPPH (2,2-Diphenyl-1-picrylhydrazyl) solution (0.070 mg mL^{-1} of methanol) – always fresh!
- blank, just methanol (instead of sample) and DPPH solution
  CAUTION: DPPH is dangerous! Use proper gloves, change immediately if contaminated! Work under ventilation hood.

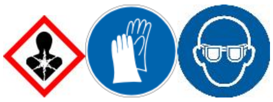

Procedure:

1. (200 µL) of DPPH solution are mixed with 100 µL of standards or different sample dilutions (25 – 75 - 100 µL ad 100 µL with H2O).
2. Incubate all samples for 1 hour at room temperature.
3. Absorbances are measured at 517 nm.
4. The radical scavenging capacity using the free DPPH radical was evaluated by measuring the decrease of absorbance at 517 nm.
5. The percentage of inhibition of samples is calculated from obtained absorbance by the equation:
   i. % inhibition = ((Abs control-Abs test)/Abs control) × 100
6. Then, curves were constructed by plotting percentage of inhibition against concentration in µg/mL. The equation of this curve allowed to calculate the IC50 corresponding to the sample concentration (biomass) that reduced the initial DPPH• absorbance of 50 %. A smaller IC50 value corresponds to a higher antioxidant activity.

STEP 3: Extraction of proteins and starch

The pellet obtained from the acetone, ethanol and methanol extraction (**STEP 2**) is air-dried on ice and subsequently used for extraction of proteins and starch.

A. *Protein Extraction Adapted from* Morgenthal et al. (2007); Nagler et al. (2015); Niessen et al. (2006)

Reagents Required:

- Urea-Extraction buffer (Urea 8M; HEPES 50mM, pH 7.8)
- Acetone and 0.5% Beta-Mercaptoethanol
- Ice-cold Acetone
- 0.1 M DTT (dithiothreitol)
- 0.1 M IAA
- 20% ACN 100mM AmBic
- 0.5M $CaCl_2$
- 10% ACN 25mM AmBic 10mM $CaCl_2$

Procedure:

Protein extraction

1. Re-suspended in 1 ml of protein extraction buffer (Urea 8M; HEPES 50mM, pH 7.8) and incubated at 4°C for 60 minutes.
2. Centrifuge sample (10.000 x g, 10 minutes, 4°C).
3. **Use Pellet for strach extractio**n -> **See STEP 3 B**
4. Transfer the supernatant into a 15 ml falcon tube and cover with 6 volumes of ice-cold Acetone with 0.5 % β-Mercaptoethanol to precipitate proteins at -20°C overnight.
5. CAUTION: β -Mercaptoethanol is toxic, corrosive and an environmental hazard! Use proper gloves, change immediately if contaminated! Work under ventilation hood.

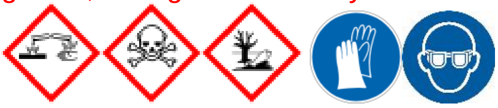

6. The next morning, centrifuge 10 min at 4°C with 4.000 x g.
7. Wash pellet three times with 1 ml ice-cold acetone always centrifuge in between and discard superanatant
8. Air dry pellets under the hood at room temperature for 5-10 min.
9. Re-dissolve pellet in 30 µl buffer containing 8 M Urea and 50 mM HEPES at pH 7.8 for protein content determination.
10. Shake on 20°C for 30 min
11. Ultrasonicate each sample for 5s
12. Centrifuge at 10.000 x g for 5 min at room temperature
13. Use Bradford protein assay to determine protein content using a standard curve of Bovine Serum Albumin standards.

Protein Reduction and Alkylation

1. Adjust Sample concentration to 10 µg protein in 8 M urea buffer.
2. Then adjust Sample to 5 mM DTT and incubate for 45 Min at 37°C and 700 rpm (reduction).
3. Add IAA to a concentration of 10 mM and subsequently incubate for 60 min at 23°C and 700rpm (alkylation).
4. To stop alkylation, adjust DTT concentration in the sample to 10 mM and incubate for another 15 min at 23°C and 700rpm.

Protein Digestion

1. For the Lys-C pre-digestion, dilute urea from 8 M to 4 M by adding one volume of 20% acetonitrile, 100 mM AmBic as well as 0.1 µg Lys-C (Promega GmbH).
2. Incubate in dark at 30°C for 5 hours with 500 rpm**.**
3. For trypsin digestion, add another volume of 10% Acetonitrile, 25 mM AmBic, 10 mM CaCl2 and 5 mM DTT to the sample resulting in a final urea concentration of 2M.
4. Add 3 µl immobilized trypsin beads (Poroszyme, Applied Biosystems) to samples and incubated overnight at 37°C.
5. centrifuge at 10.000 x g at 4°C
6. Desalt proteins with Agilent Bond Elut OMIX C18 pipette-based SPE stage tips from Agilent Technologies, according to the manufacturer's instructions
7. Dry in a vacuum concentrator (ScanSpeed MaxiVac) and store at -80°C until mreeasurement.

B. ***Extraction of starch from pellet (assay a)*** *(Nagler et al., 2015)*

Reagents Required:

- Glucose Stock (1 mM) -> 0, 25, 50, 100 µg mL^{-1})
- 0.5 N NaOH
- 1 N acetic acid (100% CH_3COOH -> 18 N)
- 5 N HCl (49.142 mL 32%[10N] HCl + 50.858 ddH_2O)
- standard-acetate-buffer (200 mL 1 N CH_3COOH, 100 mL 1 N NaOH, make volume up to 1 L)
- Tris-Glycerin-buffer (pH 7) 61 g Tris in 85 mL 5 N HCl, make volume up to 1 L with ddH_2O add 660 Glycerol and adjust pH to pH 7)
- Amyloglucosidase reagent: 10 mg Enzym in 10 ml standard-acetate-buffer
- Glucoseoxidase reagent: 15 mg Glucoseoxidase + 1.5 mg peroxidase + 5 mg o-Dianisidin-HCl in 50 mL Tris-Glycerin-buffer (pH 7) (store not longer than 12 days at 4°C)
- 80% ethanol

Procedure:

1. Wash pellets from protein exratction two times with 1 ml 80% ethanol to remove remaining urea and sugars
2. Dry at room temperature for approximately ten minutes to let solvents evaporate
3. Add 500 µl of 0.5 N NaOH to pellet and shake with 1400 rpm for 15 minutes and incubate solubilized pellets for 30 minutes at 95°C
4. add 500 µl of 1 N acetic acid and centrifuge with 13.000 rpm for 5 minutes

5. transfer 300 µl of supernatant to a new tube
6. add 300 µl of amyloglucosidase reagent and incubate at 55°C for 2 hours **(apply following steps also for each glucose standard!)**
7. after the incubation, use 25 µl of the extracts and dilute 175 µl MQ water
8. add 400 µl of glucoseoxidase and incubate for 1 hour at 30°C.
9. stop reaction by adding 800 µl of 5 N ice cold HCl,
10. Read absorbances at 540 nm.

STEP 4: Metabolite derivatization and measurement preparation:

Reagents Required:

- 40g/L CH_3ONH_2*HCl in pyridine
- N-methyl-N-trimethylsilyl-trifluoroacetamide (MSTFA)
- CAUTION: Pyridine and MSTFA are irritants and flammable! Use proper gloves, change immediately if contaminated! Work under ventilation hood.

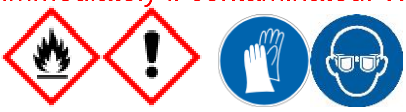

Procedure:

1. Re-dissolve dried samples, QCs and blanks in 20 µl of a 40 g l^{-1} methoxyaminhydrochlorid in pyridine and incubate at 30°C for 90 minutes.
2. Then, add 80 µl N-methyl-N-trimethylsilyl-trifluoroacetamide (MSTFA) and incubate samples at 37°C for another 30 minutes.
3. Centrifuge for 2 minutes at 14.000 x g.
4. Transferr 70 µl of the supernatant to a MS vial and apply caps with septum.
