# Supplementary material for: Rhizobium symbiosis improves amino acid and secondary metabolite biosynthesis of tungsten-stressed soybean (Glycine max)
Source: Front Plant Sci. 2024 Apr 2;15:1355136. doi: 10.3389/fpls.2024.1355136 (PMC11020092; doi:10.3389/fpls.2024.1355136)
Supplement: Supplementary file 1 [file DataSheet_1.zip › SI/Supplement_information_manuscript.docx]

Supplementary Material

***Rhizobium* symbiosis improves amino acid and secondary metabolite biosynthesis of tungsten-stressed soybean (*Glycine max*)**

Julian Preiner^1^, Irene Steccari^1^, Eva Oburger^2^, Stefanie Wienkoop^*1^,

^1^ Molecular Systems Biology Unit, Department of Functional and Evolutionary Ecology, University of Vienna, Vienna, Austria

^2^Institute of Soil Research, Department of Forest and Soil Sciences, University of Natural Resources and Life Sciences Vienna, Tulln, Austria

*** Correspondence:**Stefanie Wienkoop
stefanie.wienkoop@univie.ac.at

SI 1 | Sequential Extraction Protocol. Detailed description of extraction procedure of metabolites and proteins as well as photometric metabolite marker measurement.

SI FIGURE 1 | (A) Effective photochemical quantum yield (Y(II)) , (B) photochemical fluorescence quenching coefficient (qP) , (C) Non-photochemical quenching (NPQ) and (D) relative electron transfer rate (ETR) for PSII of dark-adapted soybean plants during the rapid light curve (RLC) measurement. Values are means ± S.E. (n = 4). Lines were smoothed by loess method; gray shades indicate 95% confidence interval. Plants (*Glycine max* cv Primus) were grown semi-hydroponically without (Ctrl.) or with 0.5 mM tungsten (W) in the nutrient solution and two different nitrogen supply regimes (N fix: inoculated with *B. japonicum*, week 1&2 0.25 mM KNO_3_ & week 3-5 zero N; N fed: week 1–5 10 mM KNO_3_).


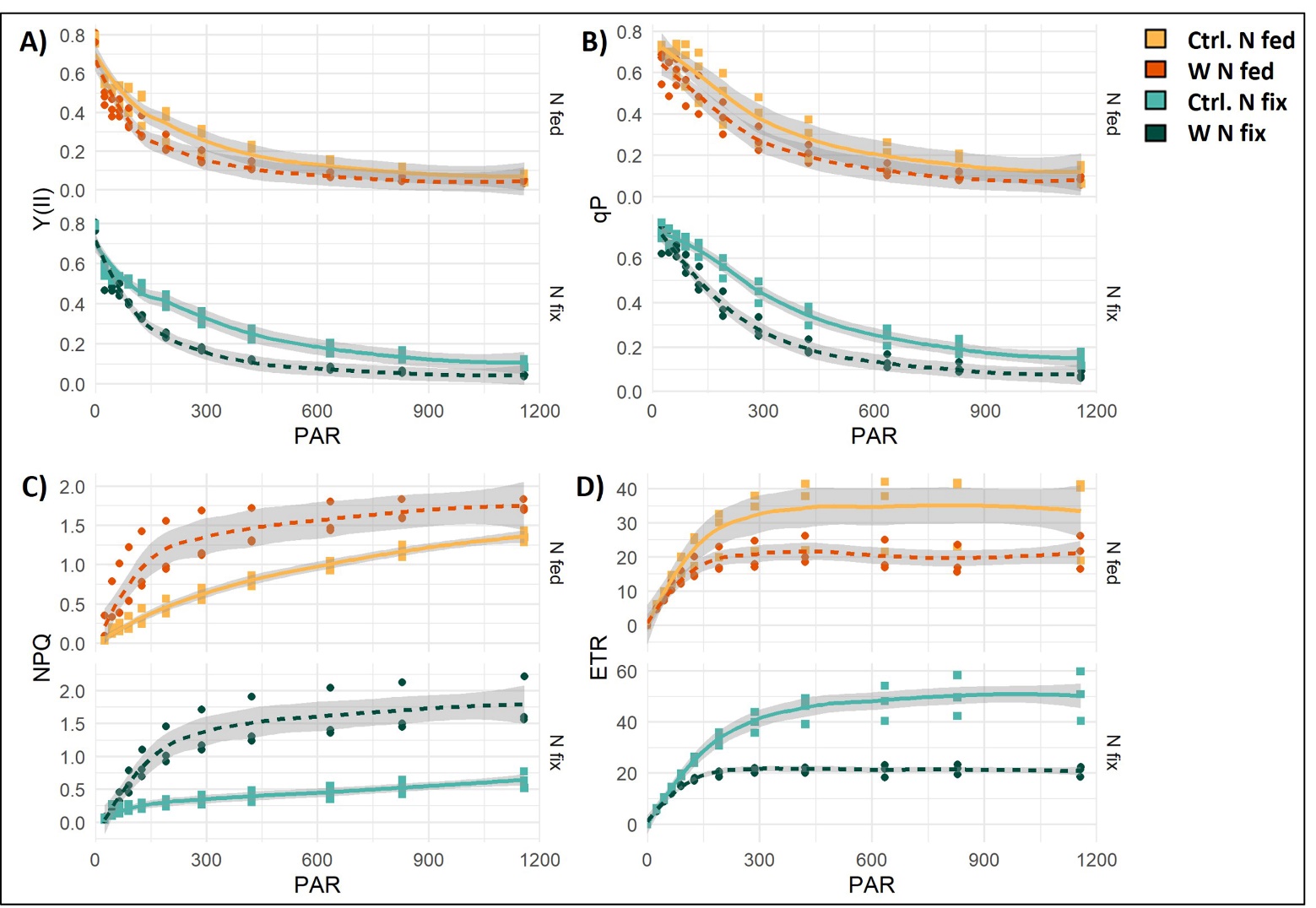


SI FIGURE 2 | PCA loadings (PC1/PC2) of leaf and root PCA including log2 transformed data including metabolite concentrations, LFQ intensities of significantly changed proteins (n=3-4, ANOVA, post hoc Tukey, p < 0.05). Figure shows a cutoff (dark grey) at ±0.065 for PC1 (A/C) and PC2 (B/D).


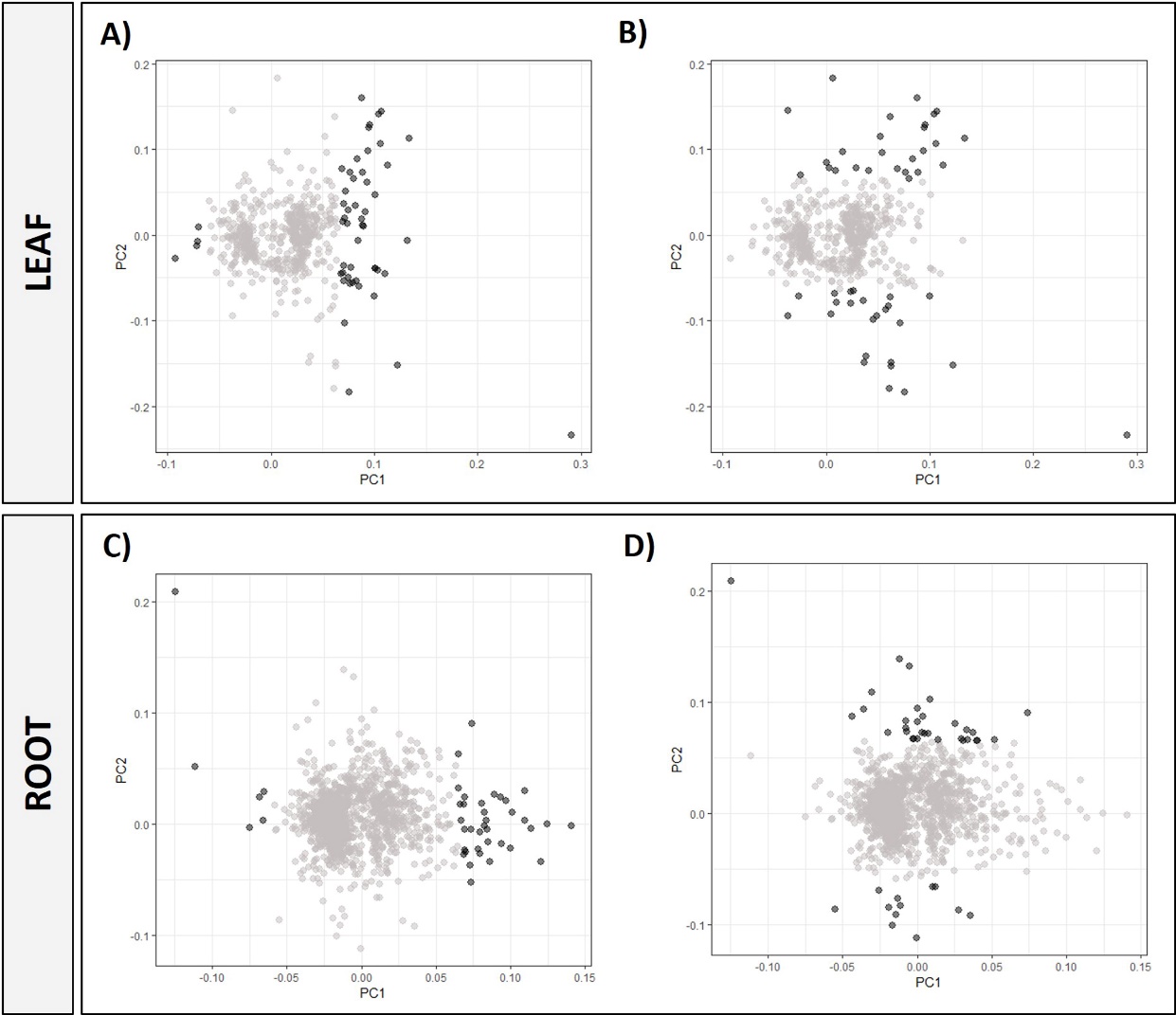


SI FIGURE 3 | Heatmap of leaf and root metabolites and proteins with PC2 loadings higher than ±0.65. Soybean plants (*Glycine max* cv Primus) were grown semi-hydroponically without (Ctrl.) or with 0.5 mM tungsten (W) in the nutrient solution and two different nitrogen supply regimes (N fix: inoculated with *B. japonicum*, week 1&2 0.25 mM KNO3 & week 3-5 zero N; N fed: week 1–5 10 mM KNO3). Colors represent z-transformed mean values (n=3-4). Letters indicate significant differences between the different tungsten treatments and nitrogen regimes (ANOVA, post hoc DGC, p < 0.05).


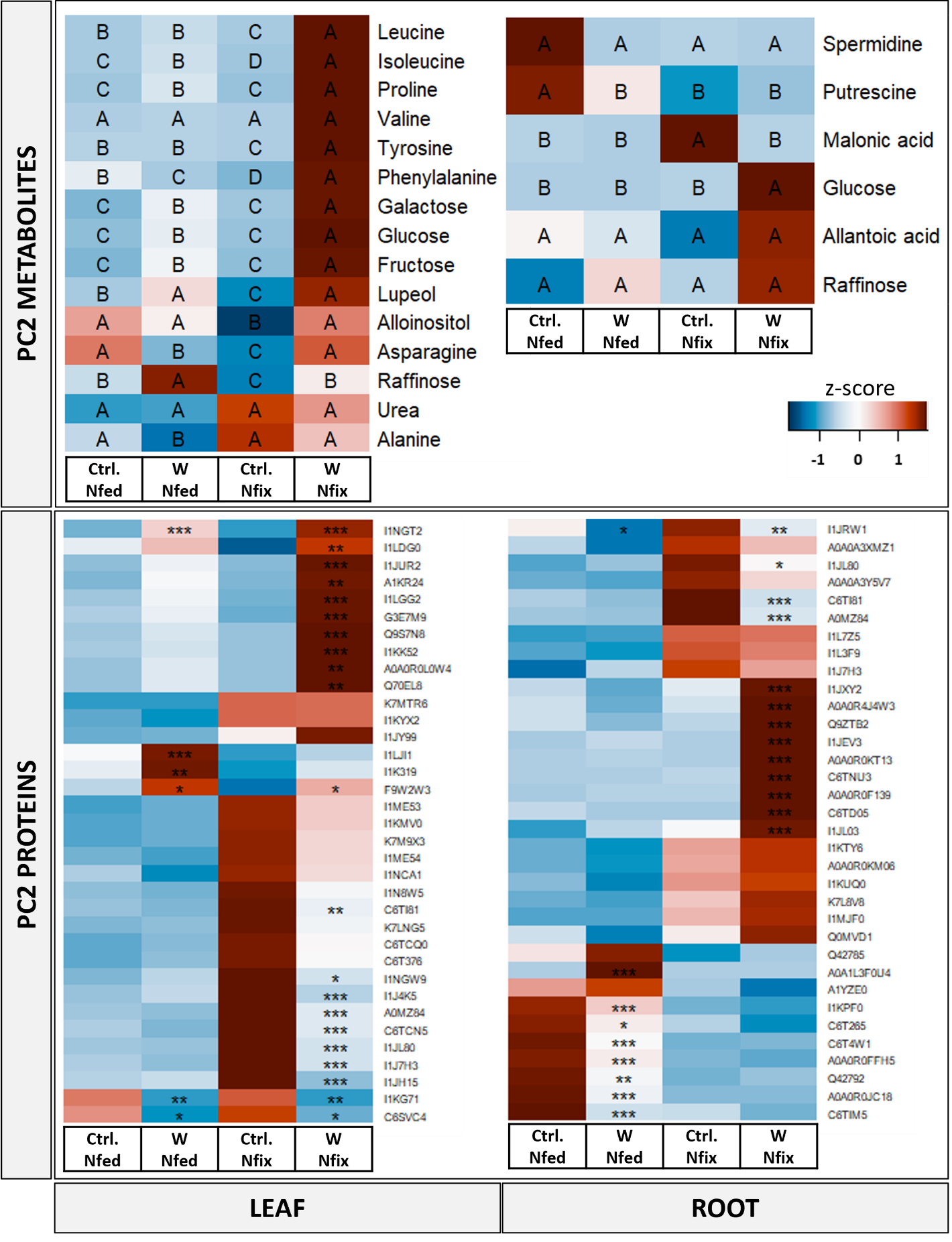


SI FIGURE 4 | Venn Diagrams of significantly changed proteins of leaves and roots. Soy bean plants (Glycine max cv Primus) were grown semi-hydroponically without (Ctrl.) or with 0.5 mM tungsten (W) in the nutrient solution and two different nitrogen supply regimes (N fix: inoculated with B. japonicum, week 1&2 0.25 mM KNO_3_ & week 3-5 zero N; N fed: week 1–5 10 mM KNO_3_). Letters indicate significant differences between the different tungsten treatments and nitrogen regimes (n=3-4, ANOVA, post hoc DGC, p < 0.05).


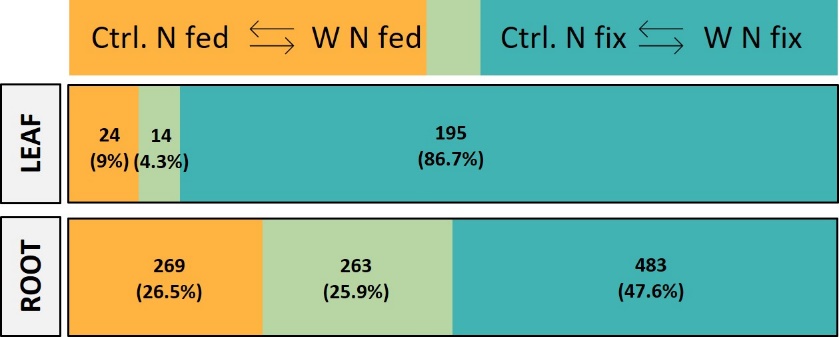


SI TABLE 1| Concentrations of photosynthetic pigments (A) as well as chlorophyll fluorescence parameters (B) of dark adapted soybean plants during the rapid light curve (RLC) measurement at a PAR of 191, 634 and 1157. Parameters shown are Fv/Fm potential quantum yield, Y(II) effective photochemical quantum yield, ETR relative electron transport rate, NPQ non-photochemical quenching, qP coefficient of photochemical quenching and qN coefficient of non-photochemical quenching. Plants (*Glycine max cv Primus*) were grown semi-hydroponically without (Control) or with 0.5 mM tungsten (W) in the nutrient solution and two different nitrogen supply regimes (N fix: inoculated with *B. japonicum*, week 1&2 0.25 mM KNO_3_ & week 3-5 zero N; N fed: week 1–5 10 mM KNO_3_). Values are means ± S.E. (n = 4). Letters indicate significant differences between the different tungsten treatments and nitrogen regimes (ANOVA, post hoc DGC, p < 0.05).

SI TABLE 2 | Leaf (A), stem (B) and root (C) concentrations, as well as root to shoot translocation factors (D) of tungsten, as well as macro- and micronutrients of soybean (*Glycine max cv Primus*) grown semi-hydroponically without (Control) or with tungsten (0.5 mM W) and two different nitrogen supply regimes (N fix: inoculated with *B. japonicum*, week 1&2 0.25mM KNO_3_ & week 3-5 zero N; N fed: week 1–5 10mM KNO_3_). Values are means ± S.E. (n = 4). Letters indicate significant differences between the different tungsten treatments and nitrogen regimes (ANOVA, post hoc DGC, p < 0.05). LOQ, limit of quantification.

SI TABLE 3 | Leaf tissue concentrations of sugars and sugar alcohols (A), amino acids (B), organic acids (C), polyamines (D) as well as further “non-QC metabolites” (E) of soybean (*Glycine max cv Primus*) grown semi-hydroponically without (Control) or with 0.5 mM tungsten in the nutrient solution and two different nitrogen supply regimes (N fix: inoculated with *B. japonicum*, week 1&2 0.25mM KNO_3_ & week 3-5 zero N; N fed: week 1–5 10mM KNO_3_). Values are means ± S.E. (n = 3-4). Letters indicate significant differences between the different tungsten treatments and nitrogen regimes (ANOVA, post hoc DGC, p < 0.05). PCA loadings for metabolites are provided for PC1-PC4.

SI TABLE 4 | Root tissue concentrations of sugars and sugar alcohols (A), amino acids (B), organic acids (C), polyamines (D) as well as further “non-QC metabolites” (E) of soybean (*Glycine max cv Primus*) grown semi-hydroponically without (Control) or with 0.5 mM tungsten in the nutrient solution and two different nitrogen supply regimes (N fix: inoculated with *B. japonicum*, week 1&2 0.25mM KNO_3_ & week 3-5 zero N; N fed: week 1–5 10mM KNO_3_). Values are means ± S.E. (n = 3-4). Letters indicate significant differences between the different tungsten treatments and nitrogen regimes (ANOVA, post hoc DGC, p < 0.05). PCA loadings for metabolites are provided for PC1-PC4.

SI TABLE 5 | List of significantly changed leaf proteins between control and tungsten (0.5 mM W, supplied as sodium tungstate) of soybean (*Glycine max cv Primus*) grown semi-hydroponically without (Control) or with 0.5 mM tungsten in the nutrient solution and two different nitrogen supply regimes (N fix: inoculated with *B. japonicum*, week 1&2 0.25mM KNO_3_ & week 3-5 zero N; N fed: week 1–5 10mM KNO_3_). Values are z-transformed means (n = 3-4). Asterisk indicate significant difference (ANOVA, Post Hoc Tukey, *p*<0.05). Proteins uniquely identified by prototypic peptides are indicated with an x.

SI TABLE 6 | List of significantly changed root proteins between control and tungsten (0.5 mM W, supplied as sodium tungstate) of soybean (*Glycine max cv Primus*) grown semi-hydroponically without (Control) or with 0.5 mM tungsten in the nutrient solution and two different nitrogen supply regimes (N fix: inoculated with *B. japonicum*, week 1&2 0.25mM KNO_3_ & week 3-5 zero N; N fed: week 1–5 10mM KNO_3_). Values are z-transformed means (n = 3-4). Asterisk indicate significant difference (ANOVA, Post Hoc Tukey, *p*<0.05). Proteins uniquely identified by prototypic peptides are indicated with an x.

SI TABLE 7 | Results of PANTHER overrepresentation test (Released 20231017) for significantly changed leaf and root proteins with highest PC1 loadings (±0.05) using a GO Ontology database DOI: 10.5281/zenodo.8436609 Released 2023-10-09 list for *Glycine max*  with 55853 gene entries as reference. Enrichment analysis was performed for biological process (BP) and molecular functions (MF). As statistical test Fisher’s exact was chosen and false discovery rate (FDR) calculated for significant enrichments.

SI TABLE 8 | Full lists with sample concentrations of quantified GC-metabolites in leaves (A) and roots (B) of soybean (Glycine max cv Primus) grown semi-hydroponically without (Control) or with 0.5 mM tungsten in the nutrient solution and two different nitrogen supply regimes (N fix: inoculated with B. japonicum, week 1&2 0.25mM KNO3 & week 3-5 zero N; N fed: week 1–5 10mM KNO3).
