# Supplementary figures and images for: Rhizobium symbiosis improves amino acid and secondary metabolite biosynthesis of tungsten-stressed soybean (Glycine max)
Source: Front Plant Sci. 2024 Apr 2;15:1355136. doi: 10.3389/fpls.2024.1355136 (PMC11020092; doi:10.3389/fpls.2024.1355136)

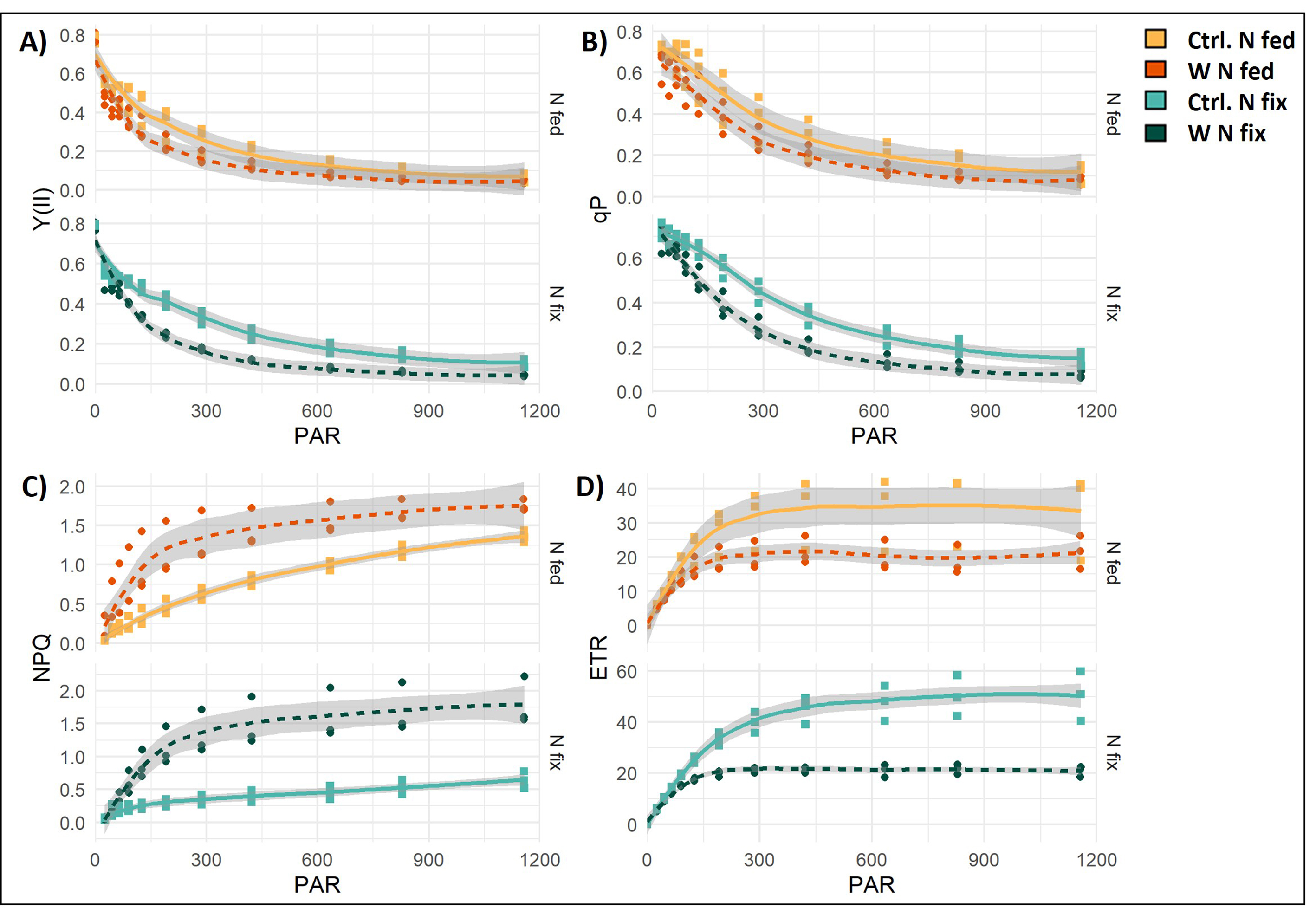

Supplement: Supplementary file 1 [file DataSheet_1.zip › SI/SI Figure 1.jpg]

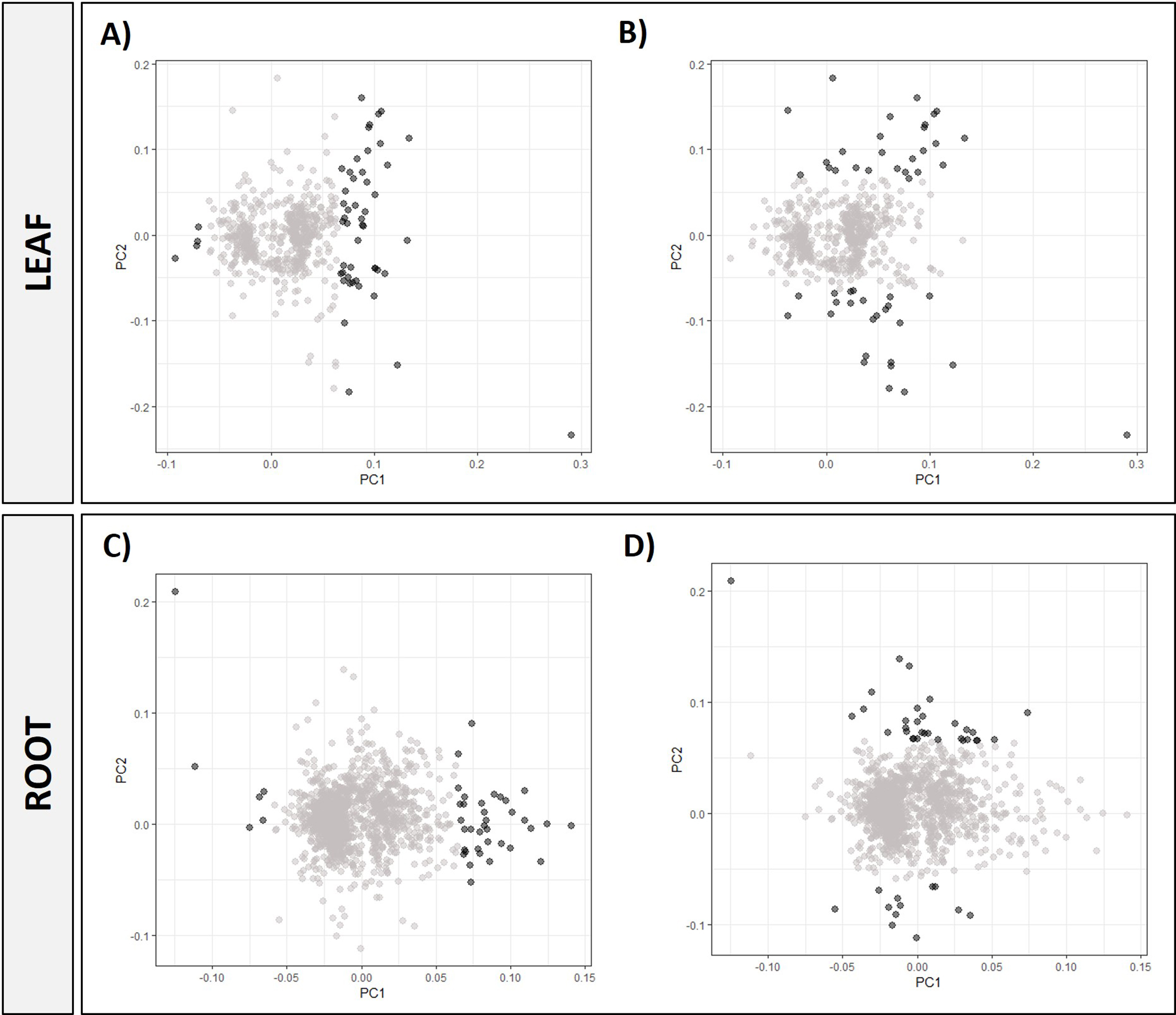

Supplement: Supplementary file 1 [file DataSheet_1.zip › SI/SI Figure 2.jpg]

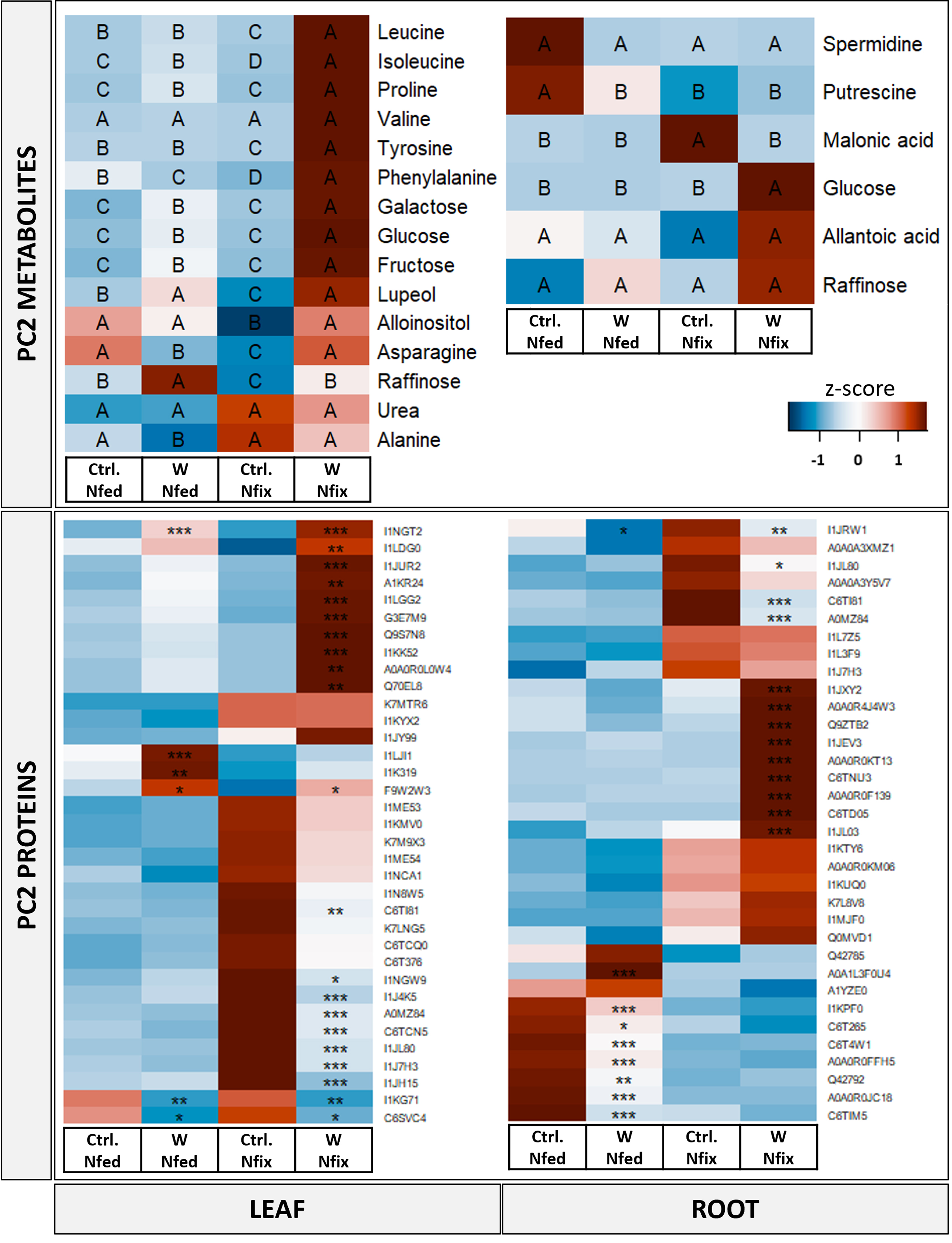

Supplement: Supplementary file 1 [file DataSheet_1.zip › SI/SI Figure 3.jpg]

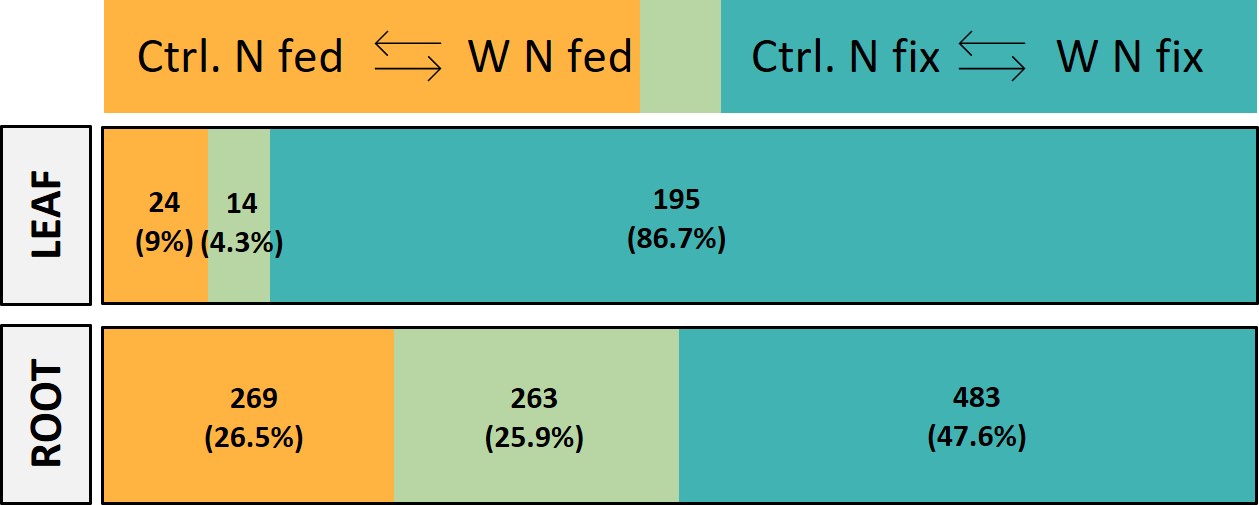

Supplement: Supplementary file 1 [file DataSheet_1.zip › SI/SI Figure 4.jpg]
